# Imaging-guided precision medicine in glioblastoma patients treated with immune checkpoint modulators: research trend and future directions in the field of imaging biomarkers and artificial intelligence

**DOI:** 10.1186/s13550-019-0542-5

**Published:** 2019-08-20

**Authors:** Mathieu Sinigaglia, Tarek Assi, Florent L. Besson, Samy Ammari, Myriam Edjlali, Whitney Feltus, Laura Rozenblum-Beddok, Binsheng Zhao, Lawrence H. Schwartz, Fatima-Zohra Mokrane, Laurent Dercle

**Affiliations:** 1 Department of Imaging Nuclear Medicine, Institut Claudius Regaud—Institut Universitaire du Cancer de Toulouse—Oncopole, Toulouse, France; 2 Département de médecine oncologique, Gustave Roussy, Université Paris-Saclay, 94805 Villejuif, France; 3 Department of Biophysics and Nuclear Medicine, Bicêtre University Hospital, Assistance Publique-Hôpitaux de Paris, 78 rue du Général Leclerc, 94275 Le Kremlin-Bicêtre, France; 4 IR4M–UMR 8081, CNRS, Université Paris Sud, Université Paris Saclay, Orsay, France; 5 Département d’imagerie médicale, Gustave Roussy, Université Paris-Saclay, 94805 Villejuif, France; 6 INSERM U894, Service d’imagerie morphologique et fonctionnelle, Hôpital Sainte-Anne, Université Paris Descartes, 1, rue Cabanis, 75014 Paris, France; 7 Department of Radiology, New York Presbyterian Hospital—Columbia University Medical Center, New York, NY 10039 USA; 8 Service de Médecine Nucléaire, AP-HP, Hôpital La Pitié-Salpêtrière, Sorbonne Université, 75013 Paris, France; 9 Département d’imagerie médicale, CHU Rangueil, Université Toulouse Paul Sabatier, Toulouse, France; 10 UMR1015, Institut Gustave Roussy, Université Paris Saclay, 94800 Villejuif, France

**Keywords:** Gliblastoma, Immunotherapy, Artificial Intelligence, Radiomics, Imaging, RANO, iRANO, PET, MR, Nivolumab, Pembrolizumab, Pidilizumab, Durvalumab

## Abstract

Immunotherapies that employ immune checkpoint modulators (ICMs) have emerged as an effective treatment for a variety of solid cancers, as well as a paradigm shift in the treatment of cancers. Despite this breakthrough, the median survival time of glioblastoma patients has remained at about 2 years. Therefore, the safety and anti-cancer efficacy of combination therapies that include ICMs are being actively investigated. Because of the distinct mechanisms of ICMs, which restore the immune system’s anti-tumor capacity, unconventional immune-related phenomena are increasingly being reported in terms of tumor response and progression, as well as adverse events. Indeed, immunotherapy response assessments for neuro-oncology (iRANO) play a central role in guiding cancer patient management and define a “wait and see strategy” for patients treated with ICMs in monotherapy with progressive disease on MRI. This article deciphers emerging research trends to ameliorate four challenges unaddressed by the iRANO criteria: (1) patient selection, (2) identification of immune-related phenomena other than pseudoprogression (i.e., hyperprogression, the abscopal effect, immune-related adverse events), (3) response assessment in combination therapies including ICM, and (4) alternatives to MRI. To this end, our article provides a structured approach for standardized selection and reporting of imaging modalities to enable the use of precision medicine by deciphering the characteristics of the tumor and its immune environment. Emerging preclinical or clinical innovations are also discussed as future directions such as immune-specific targeting and implementation of artificial intelligence algorithms.

## Background

Despite advances in treatment strategies, the prognosis for glioblastoma patients remains poor, with a median survival of around 2 years. The poor prognosis of glioblastoma patients can be attributed to their resistance to current therapeutic approaches [1]. Hence potential synergistic associations are investigated by combining existing treatments to target two hallmarks of glioblastoma: intra-tumoral heterogeneity and immunosuppressive microenvironments. Early response assessments are therefore crucial considering the poor prognosis but the state-of-the-art is complex as several combination therapies are being actively investigated.

Thousands of patients with glioblastoma recruited into international clinical trials (Table 1, 3604 pts) are currently being treated with immune checkpoint modulators (ICMs). ICMs restore the immune system’s capacity to eradicate tumors by inhibiting the immune suppressive capabilities of pathways such as CTLA-4, PD-1, and PD-L1 [2]. ICMs have advanced to the forefront of treatment of solid tumors but without leading to an impact on outcome in patients with glioblastoma in comparison with other tumors such as melanoma. Hence, they are currently used only in combination with other molecules such as chemotherapy, targeted molecular agents, vaccines, or radiotherapy.

**Table 1:**
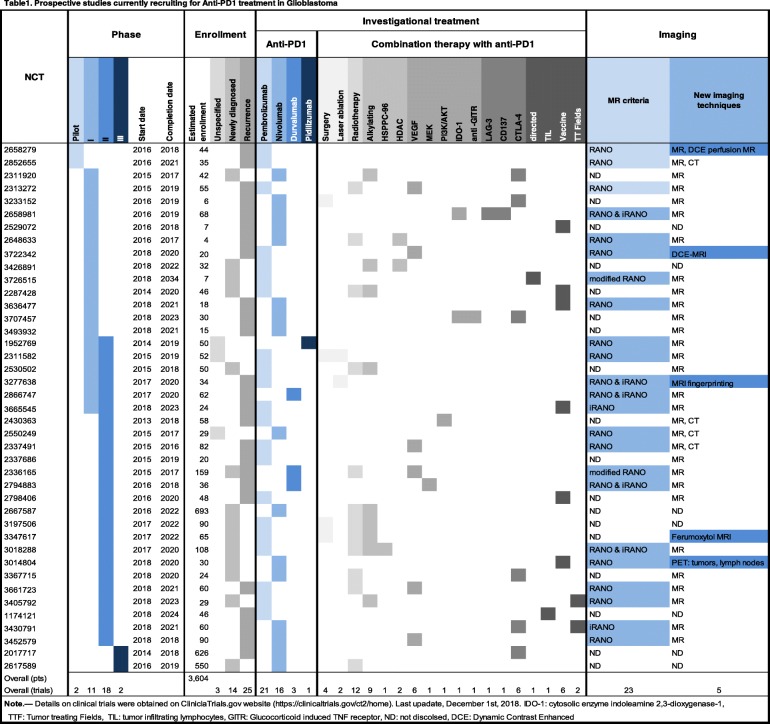
Prospective studies currently recruiting for Anti-PD1 treatment in Glioblastoma

Note: Details on clinical trials were obtained on ClinicalTrials.gov website (https://clinicaltrials.gov/ct2/home). Last upadate, December 1, 2018. *IDO-1* cytosolic enzyme indoleamine 2,3-dioxygenase-1, *TTF* Tumor treating Fields, *TIL* tumor infiltrating lymphocytes, *GITR* Glucocorticoid induced TNF receptor, *ND* not discolsed, *DCE*: dynamic contrast-enhanced

Response evaluation is intrinsically challenging in glioblastoma patients. Experts from the RANO working group have defined a compelling solution to solve (in part) imaging challenges related to chemoradiation with temozolomide (pseudoprogression) and antiangiogenic therapy (pseudoresponse): the response assessment for neuro-oncology (RANO) criteria. However, evaluating the efficacy of ICMs is a paradigm shift because ICMs trigger new imaging patterns of tumor response and progression. Experts defined a MRI-guided strategy in patients treated with ICMs in monotherapy referred to as Immunotherapy RANO (iRANO) criteria [3]. In progressive patients, a “wait and see strategy” is recommended and progression needs to be confirmed by active follow-up. However, a recent survey of 220 centers in Europe demonstrated that only a minority of centers (27%) use RANO criteria, while the majority prefers to undertake qualitative assessments. This lack of quantitative assessments demonstrates the need for standardized evaluations and the development of quantitative algorithms for robust response assessments [4].

**Fig. 1:**
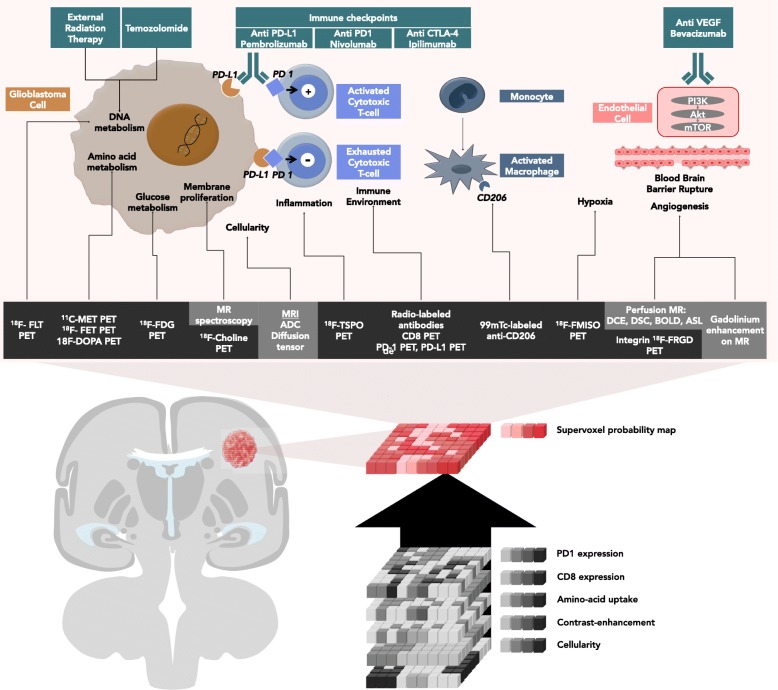
Imaging of actionable molecular pathways in patients with glioblastoma: the concept of supervoxels. Imaging allows non-invasive evaluation of the action of immune checkpoint modulators in patients with glioblastoma. Currently, most clinicians perform a visual and qualitative assessment. Alternatively, artificial intelligence can be trained to extract imaging biomarkers by measuring the signal in each unique voxel of a region of interest provided by each imaging technique. Ultimately, artificial intelligence can resume the information provided by multiple voxels from multiple imaging modality to provide one single quantitative probability map using supervoxels (synthetic summary of all voxels from the same volume of interest using different imaging modalities)

This review will discuss which imaging studies are used in ongoing clinical trials (Table 1) and what nuclear medicine specialists and radiologists should be looking for and reporting when interpreting the efficacy of ICM in monotherapy and in combination therapy. Different approaches will be described. First, standard of care imaging techniques provide non-immune-specific imaging biomarkers, which are currently widely used in routine clinical work-ups. Second, breakthroughs in biomedical engineering allow targeting immune-specific biomarkers explored in preclinical studies. Third, artificial intelligence can be trained to identify radiomics signatures by data mining standard of care MRIs. Fourth, synthetic metrics such as supervoxel [5] could capitalize on and combine these three approaches, thereby redefining medical imaging as a comprehensive and quantitative decision tool. (Fig. 1, Tables 2 and 3).

**Table 2:** MRI imaging biomarkers for assessment of the immune and tumor environment of glioblastoma

Hallmark	Threshold	Advantages	Limitations
Cellular proliferation	*MRS*: ↑Chomax, ↑Chomean, ↓NAA, ↓Cr, ↑mI, ↓NAA/Cr, ↑Cho/Cr, ↑Cho/NAA ratio *MRI*: ↓ADC	Specificity	Tumor heterogeneity Low sensitivity (mM) No specific patterns Acquisition time (MRS) Peripheral lesions (MRS: pitfalls with bone and skin) No absolute reference value (ADC)
Membrane proliferation	*MRS*: ↑Chomax, ↑Chomean, ↑Cho/Cr, ↑Cho/NAA ratio		Low sensitivity (mM)
Structural complexity	↑Diffusion kurtosis imaging ↓ Fractional anisotropy (brain fibers)	Specificity Sensitivity	Availability Still experimental
Aminoacid metabolism	–	–	–
Glucose metabolism	*MRS*: ↑free lipids	–	Pitfalls: lymphoma, lactates (TE = 35 ms)
Angiogenesis	↑Ktrans on DCE-MR (permeability) ↓BOLD fMRI signal	Level of evidence	Software Steroids Non specific
Perfusion	*DSC MRI*: ↑CBV↑CBF↑rCBV > 4 ↑Relative CBV on DCE-MR	Robust software	Normalization is required Operator dependent Nonspecific of gliomas
Invasiveness	↑FLAIR ↓ADC (except in the edema) ↓FA (experimental)	Sensitivity	Non-standardized Operator dependent Normalization is required
Hypoxia	*MRS*: lactate > 0 (no lactate accumulation in healthy tissue) ↓19F-MRI	Specificity	Non-standardized
Necrosis	↓DWI ↓ADC *MRS*: lipids > 0 (TE = 35 ms), no lipid accumulation in healthy tissue, Lactate > 0	Specificity	Non-standardized
Edema	↑ADC ↑T2FLAIR	Sensitivity	Specificity
Infiltration of cytotoxic T cells	–	–	–
Anergy of T cells	–	–	–
Activated microglia	–	–	–

Note: [6]. *MRS* magnetic resonance spectroscopy, *Chomax* maximum concentration of choline-containing compounds, *Chomean* mean concentration of choline-containing compounds, *Cr* creatinine, *mI* myoinositol, *NAA N*-acetyl-aspartate, *BBB* blood-brain barrier, *CBV* cerebral blood volume, *CBF* cerebral blood flow, *rCBV* related CBV, *FLAIR* fluid-attenuated inversion recovery, *ADC* apparent diffusion coefficient, *FA* fractional anisotropy. *BOLD* blood oxygenation level dependent, *fMRI* functional magnetic resonance imaging, *TE* EchoTime (ms), ↓ decrease, ↑ increase

**Table 3:** PET imaging biomarkers for assessment of the immune and tumor environment of gliobastoma

Hallmark	Threshold	Advantages	Limitations
Cellular proliferation	↑18F-FLT	Correlated to Ki-67 High sensitivity (nM) Absolute quantification	Does not cross the intact blood-brain barrier (BBB) High cortical background activity Low specificity Challenging production
Membrane proliferation	↑18F-choline	High sensitivity (nM) Absolute quantification Radiation necrosis vs. recurrence	Does not cross the intact BBB Inflammation vs. Tumor tissue High cortical background activity Availability
Structural complexity	–	–	–
Aminoacid metabolism	↑11C-methionine ↑18F-FET ↑18F-FDOPA	Cross the intact BBB Specificity	Half-life (11C- methionine = 20 min) Availability
Glucose metabolism	↑18F-FDG	Availability Cross the intact BBB No side effects	High cortical background activity Non-specific: inflammation vs. tumor
Angiogenesis	↑18F-RGD	Marker for αVβ3 expression	Primarily an experimental application
Perfusion	↑15O-H2O	Quantification in mL/100 g per min	Availability Time and cost consuming
Invasiveness	–	–	–
Hypoxia	↑18F-FMISO ↑18F-FAZA ↓15O-H2O	Identification of radiation resistant areas	Primarily experimental application
Necrosis	–	–	–
Edema	–	–	–
Infiltration of cytotoxic T cells	↑18F-FHBG	Track HSV1-tk reporter gene expression (cytotoxic T cells)	Preclinical experimental application
	↑ 89Zr-PEGylated-anti-CD8-VHH	Track CD8+ T cells	Primarily experimental application
	↑68Ga-DOTA-D-Phe1-Tyr3-Octreotide	Activated immune cells	Primarily experimental application
Anergy of T cells	↑PD-1 or PD-L1	Prediction of the effectiveness of anti-PD1	Still experimental on mouse tumor models
Activated microglia	↑TSPO (immuno-PET)		Nonspecific: tumor vs. neuro-inflammation

^*18*^*F-FLT* ^18^F-fluorothymidine, *BBB* blood-brain barrier, *18F-FDG* ^18^F-fluorodeoxyglucose, ^*18*^*F-FET* ^18^F-fluoroethyltyrosine, ^*11*^*C-MET* 11C-methionine, ^*18*^*F-RGD* ^18^F-arginine-glycine-aspartic acid, ^*18*^*F-FMISO* ^18^F-fluoromisonidazole, ^*18*^*F-FHBG* ^18^F-fluoro-3-(hydroxymethyl)butylguanin, ↓ decrease, ↑ increase

This report aims to provide a structured approach for standardized selection of imaging modalities to enable a precision medicine approach by deciphering the characteristics of the tumor and its immune environment. Furthermore, this review addresses challenges faced by radiologists evaluating patients treated with ICMs: the evaluation of ICMs in combination therapies, new patterns of response (i.e., pseudoprogression, hyperprogression and abscopal effect), the accuracy of alternative imaging metrics to differentiate tumor progression from delayed responses or from therapy-induced inflammation.

## Rationale for ICM in combination therapy

The current standard of care for glioblastoma treatment involves surgical resection followed by a 6-week course of radiation therapy with 60 Gy delivered in 30 fractions [7]. The oral alkylating agent Temozolomide (TMZ) is used as concomitant and adjuvant chemotherapy at a dose of 75 mg/m^2^ daily, throughout the radiation therapy [8]. In cases of disease recurrence after this protocol, the treatment may involve a new surgery, new radiation therapy or the use of bevacizumab (antibody targeting VEGF) [9].

The frequency and severity of glioblastoma explain how critical the optimization of treatment strategies is [10]. Glioblastoma is indeed the most common primary malignant brain tumor in adults and the median survival with current treatment strategies is 15 months[11]. An even poorer prognosis is observed with male patients [12] older than 50 years [13] with neurological or general symptoms [14].

There is a strong rationale for the use of ICM. Glioblastoma cells [15, 16] escape immune surveillance by creating an immune-suppressive environment [17], which is further promoted by central nervous system immune isolation, blood-brain barrier protection[18], the low activity of the major histocompatibility complex, and the low quantity of antigen presenting cells. ICMs aim to restore tumor elimination (Fig. 1) through the activation of anergic T lymphocytes. Immune cells are indeed able to migrate across the blood-brain barrier to reach cervical lymph nodes and present tumor antigens.

The limited efficacy of the standard of care therapies, as well as ICMs in monotherapy [19] (Table 1), have led to exploration of synergistic therapeutic combinations (Table 1) involving ICMs, radiotherapy, and systemic therapy. The rationale for radiotherapy is that it improves the response of tumors to ICMs by modulating the expression of molecules on the surface of tumor cells (e.g., major histocompatibility complex-1, calreticulin, PD-L1 [20]), increasing the secretion of pro-inflammatory cytokines (e.g., interferon gamma) and enhancing the recruitment of immune cells (e.g., it releases tumor antigens into the circulation, decreases the tumor interstitial fluid pressure [21], and activates CD8 T-Cells [22]). Alternatively, combinations with systemic therapy are also being actively investigated. This is exemplified by antiangiogenic drugs such as bevacizumab, which modulates immune response, the number of active T-cells, and the maturation of dendritic cells [23–25].

## Immune-related patterns of response and progression

Because of the distinct mechanisms of ICMs that restore the immune system’s anti-tumor capacity, unconventional immune-related phenomena are encountered in terms of tumor response and progression, and adverse events.

Pseudoprogression defines a transitory progression in tumor size or metabolism and can mislead the evaluation of cancer treatment efficacy. The pseudoprogression can be due to either delayed therapeutic efficacy or immune cell infiltration. These phenomena constrain clinicians to a *wait and see* strategy in case of appearance of growing disease, since tumor growth or new lesions do not preclude clinical benefit, treatment efficacy, and long-term survival. High rates of disease pseudoprogression are expected in glioblastoma patients treated with ICM in combination with standard-of-care therapies (e.g., radiotherapy), since pseudoprogression already occurs in up to 30% of glioblastomas treated with standard-of-care therapies and up to 10% [26, 27] of solid tumors treated with ICMs. Several lessons can be learned from classical treatments [28]. First, the only validated diagnostic criterion of a pseudoprogression is the stability or improvement over time. This strategy is problematic in glioblastoma patients given their short life expectancy[3]. Second, MRI changes observed in pseudoprogression are not specific (the increase in contrast enhancement and signal abnormalities on T1, T2, and Flair sequences). Third, in the majority of cases, pseudoprogression occurs within the first 12 weeks after completion of chemoradiation [29]. Consequently, alternative imaging criteria are needed (Fig. 1).

**Fig. 2:**
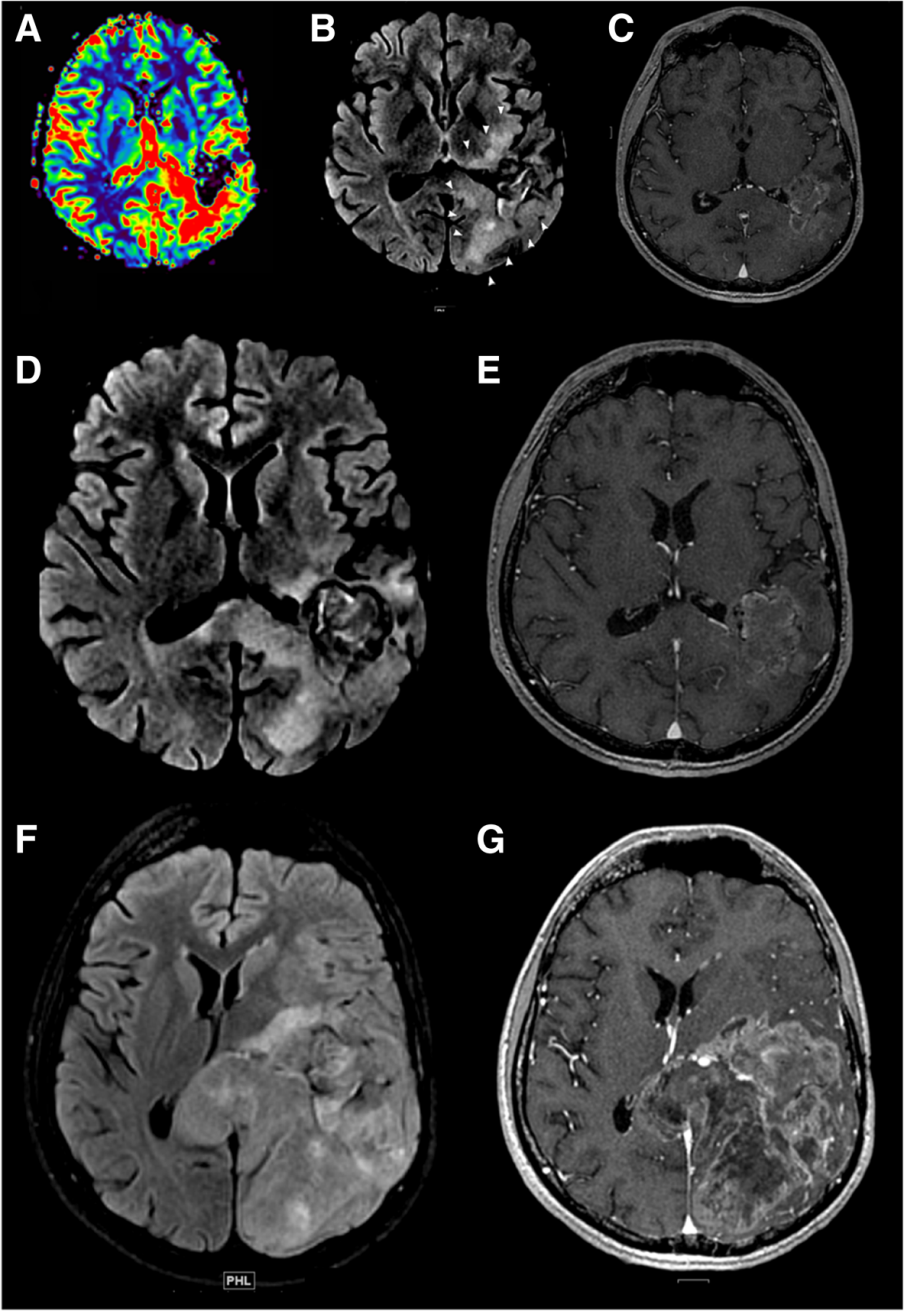
Detection of a potential hyperprogression in a patient with glioblastoma. This case illustrates the potential risk of hyperprogression. Imaging of an 18 year old patient with a diagnosis of glioblastoma treated with anti-PD-1. MRIs were obtained at 3-month intervals (baseline, **a**–**e**; 3 months, **f**, **g**). **a**–**e** Baseline T1 post-contrast MRI prior to immunotherapy and re-gamma knife therapy demonstrating an enhancing lesion with increased perfusion. **f**, **g** MRI post-initiation of immunotherapy showing fast interval growth of the lesion, as well as a life-threatening mass effect. This case illustrates the potential life-threatening local complications of hyperprogression

Hyperprogression defines an acceleration of tumor growth after the initiation of ICM therapy, as compared to the period before treatment initiation used as a reference. Hyperprogression was reported in 9–29% of patients with solid tumors and was associated with a shorter overall survival [30] (Fig. 2). An idiosyncratic effect of ICMs is suspected [31].

The abscopal effect defines the occurrence of an objective response outside of the radiation field [32] when radiation therapy is combined with ICM. The abscopal effect is triggered by several factors such as (1) the modulation of the expression of molecules on the surface of tumor cells [20], (2) increased expression of pro-inflammatory cytokines, and (3) enhancement of the recruitment of immune cells [21]. The role of abscopal effects related to ICMs in glioblastoma patients/in the CNS needs to be investigated.

Pseudoresponses define a transitory radiographic response due to an action on blood vessel permeability rather than an anti-tumor effect. Pseudoresponses occur in antiangiogenic therapies and not in treatment with ICMs in monotherapy [3, 33]. In antiangiogenic therapies, the RANO criteria require a radiographic response to persist for more than 4 weeks: a rapid radiographic response can be observed in up to 60% of patients and is not related to increased survival.

Immune related adverse events (iRAE) can occur theoretically at any site and at any time in patients treated with ICMs. In patients with glioblastoma, the radiologists should be aware that systemic ICM therapies are expected to trigger iRAE most frequently at specific sites such as lung, mediastinum lymph nodes (sarcoidosis-like), colon (enterocolitis), glands (hypophysitis, thyroiditis), liver (hepatitis), pancreas (pancreatitis), and joints (arthralgia). Life-threatening iRAE should be suspected in case of occurrence of pneumonitis and colitis. Medical imaging detects 74% of irAE in patients treated with anti-PD1 and guides patients and their health care providers towards specific management [34].

## Current guidelines

**Fig. 3:**
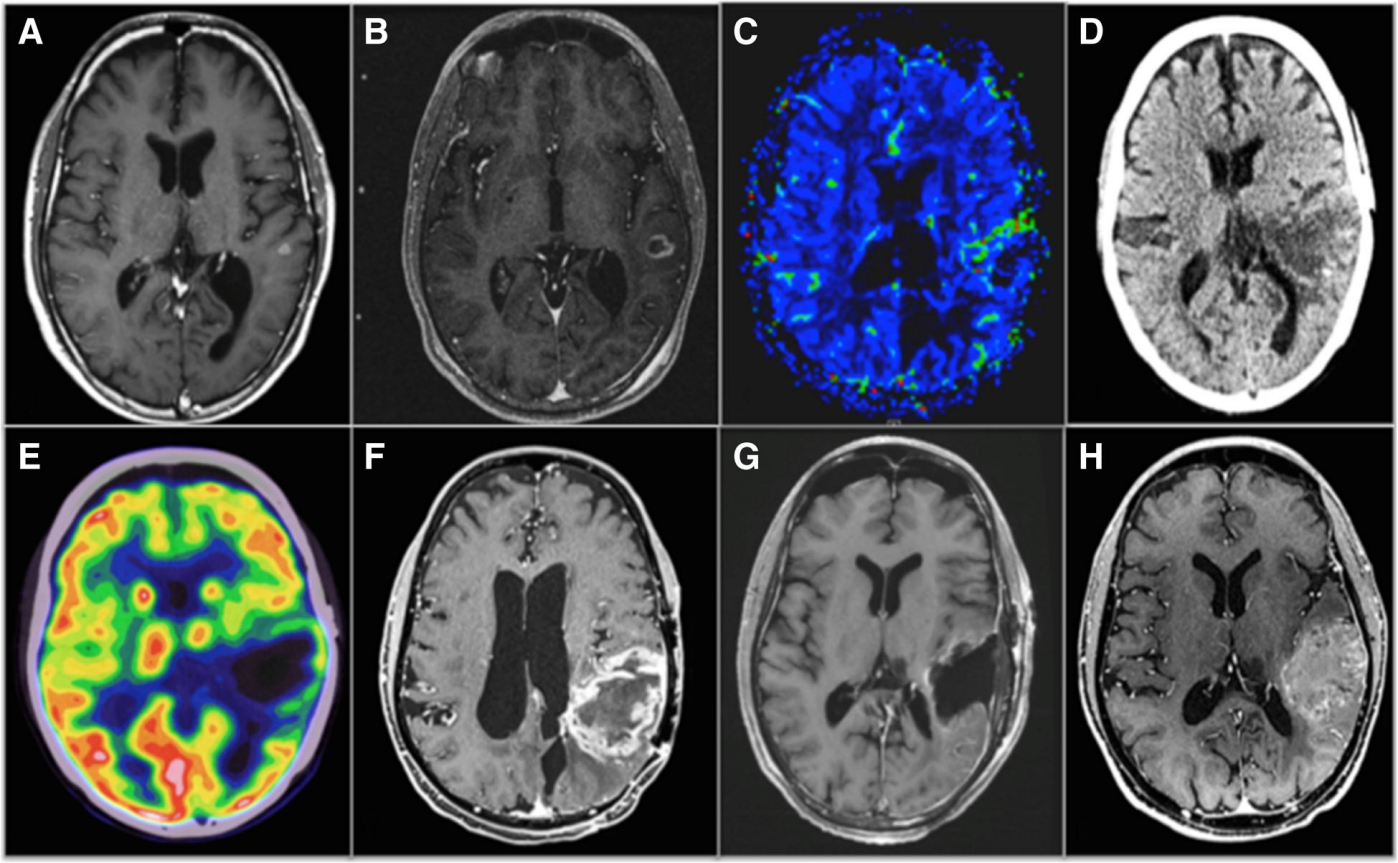
Multimodal image-guided management in a PD-1, PD-L1, TILs glioblastoma. This case illustrates the potential interest of pre-immunotherapy immuno-PET imaging biomarkers since the immune escaping environment (i.e., pathology was negative for PD-1, PD-L1 and, tumor infiltrating lymphocytes) explaining the insensitivity of this patient to immunotherapy was demonstrated only on the pathology post-resection at the end of immunotherapy. Existing imaging techniques demonstrated treatment insensitivity (**a**–**h**) but were not able to decipher the immune contexture for an early prediction of outcome. Imaging of a patient with recurrent glioblastoma in the left parietal lobe treated with combined immunotherapy (nivolumab) and re-gamma knife. MRIs were obtained at 3-month intervals. **a** Baseline T1 post-contrast MRI prior to immunotherapy and re-gamma knife therapy demonstrating a 6 × 5 mm enhancing lesion in the left parietal lobe. **b** MRI post-initiation of immunotherapy and pre-re-gamma knife therapy showing interval growth of the lesion. **c** MRI perfusion demonstrating growth and increased flow along the anterior margin of the tumor. **d**, **e** PET/CT demonstrating continued growth and increased FDG activity along the margin of the lesion. **f** Subsequent MRI demonstrating significant growth, increased peripheral nodular enhancement, and central necrosis. **g** Post-contrast MRI post-resection showing mild non-specific enhancement around the resection margin. **h** Follow-up MRI 7 months after resection demonstrating progression of disease

There is a crucial need for defining the optimal imaging-guided strategy in glioblastoma patients treated with ICMs, both in monotherapy and combination therapy. The only existing guideline was proposed by the RANO working group (iRANO criteria) and concerns response assessment using contrast-enhanced MRI in patients treated with ICMs in monotherapy (Fig. 3) [3].

On MRI, iRANO criteria recommend a “wait and see” strategy in patients with a radiological progression within 6 months after initiating ICMs in monotherapy [3] due to the pseudoprogression phenomenon. A radiological progression is defined by a worsening of clinical status (i.e., neurological symptoms and consumption of corticoids), an increase in the size of contrast enhancement of target lesions, or an apparition of new lesions. Strikingly, the management of combination therapies and hyperprogression was not discussed by the RANO working group.

While MRI is the current standard of care for staging and response assessment, guidelines [35] increasingly recommend, in addition, the use of amino acid positron emission tomography (PET) to detect viable tumor tissue, tumor delineation (estimation of true tumor extension in low- and high-grade gliomas), selection of the best biopsy site (stereotactic biopsy guiding), non-invasive tumor grading (combination of dynamic 18F-FET-PET and diffusion MRI), therapy planning (defining the true tumor volume to be treated), treatment monitoring (response assessment to locoregional chemo- and radiotherapy), and early detection of residual tumor after surgery. However, the role of amino acid PET in ICM response assessment remains unaddressed.

## Limitations of conventional non-immune-specific MRI biomarkers

In patients with glioblastoma, diagnosis and response assessment rely on various imaging techniques not designed for ICM monitoring (Table 2) which are, therefore “non-immune-specific.” MRI sequences include post contrast T1- and T2-weighted images, diffusion and perfusion imaging, and proton magnetic resonance spectroscopy.

### Cellular density: diffusion-weighted imaging

MRI measures cellular density through the apparent diffusion coefficients (ADCs) on diffusion-weighted images (DWIs), measuring itself the random diffusion of water molecules (Brownian motion) in biological tissues [36]. The paradigm in cytotoxic treatment is that a decrease in ADC reflects degradation of cellular integrity by necrosis or edema [37, 38] and predicts treatment efficacy [39]. In patients treated with ICM, the interpretation of ADC is not straightforward. Indeed an increase in the volume of tissue with intermediate ADC predicts efficacy [40] while an inflammatory cell swelling and macrophage recruitment can decrease ADC [40]. Imaging biomarkers derived from ADC were also leveraged to guide dendritic cell immunotherapy (minimum ADC [41] and percentage of voxels with decreasing ADC [42]). Therefore, the role of ADC in predicting response to ICM combined with angiogenesis inhibitors remains to be elucidated considering that each one of this two treatments in monotherapy have an opposite effect on ADC [39, 43].

### Cellular density: fractional anisotropy (FA)

Fractional anisotropy is used in clinical research to estimate tissue viability and brain fiber integrity [44]. Interestingly, changes in fractional anisotropy appraise treatment efficacy and can occur as soon as 1 day after the initiation of cytotoxic chemotherapies [38]. Although FA has not been investigated in ICMs, a recent study on brain metastases has shown that FA reflects immune microenvironment activity. This could be leveraged in patients treated with ICM since higher T-cell infiltration co-localizes with white matter disruption and a decrease in anisotropic diffusion [45]. A current drawback is that there are significant inter-observer and inter-structure variations in fractional anisotropy [46].

### Membrane proliferation

Magnetic resonance spectroscopic imaging (MRSI) can estimate the concentration of a subset of specific brain metabolites such as choline and creatinine. This technology is used to diagnose tumor tissue which is characterized by a high concentration of choline metabolites and low creatinine metabolites [37]. Creatinine reflects cellular integrity and is usually used to balance the lack of specificity of evaluating choline concentration alone. The inherent limitation of MRSI for the assessment of ICM is that membrane proliferation is a nonspecific process observed in neoplastic and inflammatory diseases. Nevertheless, a pivotal report demonstrated that choline imaging was more representative of the tumor volume than gadolinium enhancement in glioblastoma treated by intralesional immunotherapy [47]. There is therefore a rationale suggesting that a lesion with gadolinium enhancement without increased membrane proliferation suggests a “flare phenomenon” which usually resolves within 3 months [48]. Clinical value in systemic ICMs has to be evaluated.

### Angiogenesis and perfusion

Glioblastoma is among the most vascularized solid tumors. A wide range of advanced MRI sequences allows a comprehensive analysis of tumor angiogenesis: gadolinium contrast enhancement [49], perfusion-weighted imaging [36], dynamic contrast-enhanced magnetic resonance imaging (dynamic T1-weighted approach), arterial spin-labeling, or T2-weighted rapid echo-planar sequence (DSC-MRI). Arterial spin labeling (ASL) is a promising perfusion parameter using arterial blood proton signals after magnetic labeling when gadolinium is not usable [50, 51]. Preliminary results suggested that this technology could be used in the future to optimize the management of the combination of antiangiogenic therapies, and ICMs which are currently investigated in most clinical trials (Table 1). Typically, effective antiangiogenic therapies induce a steroid-like effect, normalize blood-brain barrier permeability, and so decrease MR enhancement [52–54]. These parameters can also be used to differentiate immune system-induced inflammation such as pseudoprogression (low cerebral blood volume) from true tumor growth (high cerebral blood volume) in glioblastoma patients treated with radiation therapy [55] or ICM [41]. Immunotherapeutics can also lead to an early increase in contrast enhancement, due to the inflammatory response [40]. In this case, pseudoprogression can be suggested if the neo-angiogenesis is absent on the perfusion sequences or the contrast enhancement is far from the initial lesion and within the radiotherapy field.

### Hypoxia

The extremely poor prognosis of glioblastoma is mostly attributed to the high percentage of hypoxic niches in the tumor microenvironment. Functional MRI (blood ixygenation level-dependent or BOLD MRI, ^19^F-MRI, electron paramagnetic resonance) [56] is used to detect hypoxia in clinical research. A decrease in the fMRI activation volumes on BOLD fMRI adjacent to a glioblastoma was observed in aberrant neo-angiogenesis, with the resultant de-coupling of blood flow from neuronal activity [57]. Recent results suggest that functional MRI could play a significant role in the monitoring of antiangiogenic therapies [58] or ICMs [59]. Additionally, clinical trials using local T cell immunotherapy (autologous primary human CD8^+^ cytolytic T lymphocytes) have demonstrated that MRI sequences can be used to detect a T-cell mediated necrosis. This pattern should be studied in depth in patients treated with systemic immunotherapy.

## MRI biomarkers under investigation

### Techniques investigated in ongoing ICM trials

#### Alternative contrast agents

Ferumoxytol is an ultrasmall superparamagnetic iron oxide used as an alternative contrast agent in patients with impaired renal function and is currently being investigated in ICMs (Table 1). It has as a unique feature, a prolonged intravascular residence time of more than 12 h because of its size and carbohydrate coating. The use of cerebral blood volume (CBV) mapping with ferumoxytol may help determine therapeutic efficacy in a variety of brain tumors by differentiating highly vascular malignant tumor tissue from treatment-related neuro-inflammation, which correlates with survival [60–62].

#### Magnetic resonance fingerprinting

MRI acquisitions are often restricted to a qualitative or “weighted” measurement and are almost never quantitative. The same tissue can have different intensities in different data sets depending on several cofounding variables (e.g., type of scanner, type of detectors). Magnetic resonance fingerprinting (MRF) takes a different approach to data acquisition, post-processing, and visualization, by using a pseudorandomized acquisition generating a unique signal evolution or “fingerprint” simultaneously representing all investigated tissue properties [63]. MRF could thus provide highly specific and quantitative images and is currently being investigated (Table 1) [64].

### Artificial intelligence-derived MRI biomarkers

**Fig. 4:**
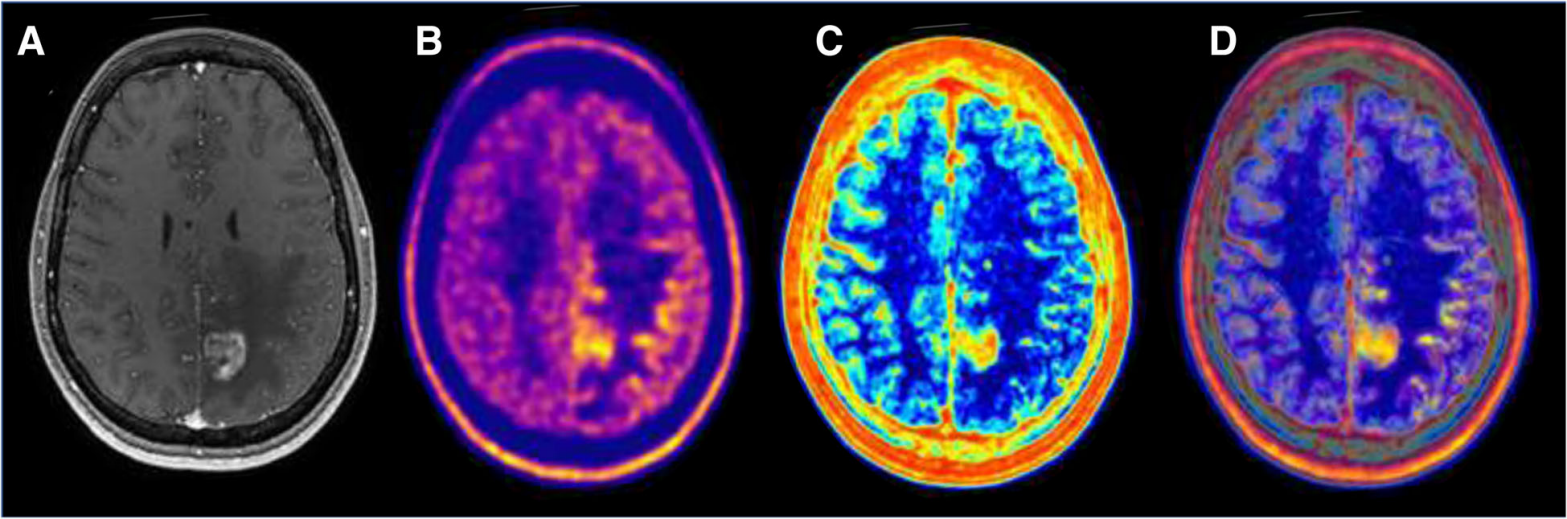
Multimodal image-guided management using artificial intelligence in glioblastoma. This case illustrates the potential interest of imaging biomarkers extracted using artificial intelligence. Imaging of a patient with glioblastoma. **a** Baseline T1 post-contrast MRI prior to therapy demonstrating an enhancing lesion. **b** Baseline 18F-Dopa PET showing an increased amino acid uptake outside of the enhancing lesion on MRI. **c** Analysis of the MRI by artificial intelligence demonstrating areas with high heterogeneity (red) and low heterogeneity in normal healthy brain tissue (blue). This map is a parametric map of local entropy computed using the baseline T1 post-contrast MRI. The only limit in the analysis of the local heterogeneity/entropy is that contours/edge/interface are always heterogeneous. **d** Fused image of the Baseline 18F-Dopa PET (**b**) and of the parametric map of local entropy (**c**)

There is a strong rationale suggesting that artificial intelligence (AI) could be used to optimize the management of patients with glioblastoma [65] (Table 4). First, radiologists’ visual assessment does not use all information available in medical images. Second, treatment monitoring and strategies are increasingly complex. Radiomics is a fast-evolving field in medical imaging consisting in the extraction of high-throughput quantitative imaging features that characterize the inner organization of a tumor. The core assumption is that medical images contain quantitative information that could be used to optimize patient’s treatments. Thus, the computer can associate specific imaging traits to tumor characteristics, prognosis, optimal treatment, or tumor response (Table 4). AI can even combine information from different imaging techniques to provide unique synthetic information analyzable by the clinician: one single quantitative probability map of “supervoxels” (Fig. 1 and 4). Theoretically, AI could be trained to identify patterns associated with responses to ICM in monotherapy or in combination. However, there are limitations to AI approaches. The major drawback is that building a reliable predictive model with AI requires a large amount of well-annotated clinical and imaging data to avoid overfitting. In the field of glioblastoma imaging, we can assume that the use of AI will therefore be first restricted to the use of MRI in standard of care therapies.

**Table 4:** Current precision diagnosis and treatment approaches using radiomics on standard of care MRI sequences in patients with glioblastoma

Year, Author	Sequence	Training and Validation set	Extracted radiomics features, selection, and statistical learning	Biologic correlation and relevance
2008, Diehn	T1, T1+ T2	T, 22 pts V, 110 pts	-10 binary imaging traits (enhancement, necrosis, mass effect, T2 edema, cortical involvement, SVZ involvement, C:N ratio, contrast/T2 ratio, T2 edema, T2 heterogeneity). - Unsupervised hierarchical clustering, Spearman rank-correlation coefficient.	- Associations between angiogenesis, tumor hypoxia, and the contrast enhancement imaging phenotype; proliferation gene-expression signature and mass effect phenotype; EGFR protein overexpression and contrast:necrosis imaging trait.
2011, Zinn	FLAIR	T, 26 pts V, 26 pts	- Quantitative models of edema/invasion, enhancing tumor, necrosis. - Comparative marker selection, ingenuity pathway analysis.	- Imaging traits associated with upregulation of mRNA involved in cellular migration/invasion (PERIOSTIN),which was seen to correlate with decreased survival.
2014, Rahman	ADC-/+ T2/FLAIR	T, 91 pts	- 6 variables extracted from histograms of apparent diffusion coefficient were measured at three times (baseline, post-treatment and change). - Cox proportional hazards model adjusted for clinical variables.	- ADC histogram analysis within both enhancing and nonenhancing components of tumor can be used to stratify for PFS and OS in patients with recurrent glioblastoma treated with Bevacizumab.
2014, Jamshidi	T1, T1+ T2 Flas	T, 23 pts	- (1) infiltrative versus edematous T2 abnormality, (2) degree of contrast enhancement, (3) necrosis, (4) supraventricular zone (SVZ) involvement, (5) mass effect, and (6) contrast-to-necrosis ratio. - Resampling statistics, analysis of variance, Pearson correlation coefficient.	- Gene-to-trait associations were found such as contrast-to-necrosis ratio with KLK3 and RUNX3, SVZ involvement with the Ras oncogene family and the metabolic enzyme TYMS, and vasogenic edema with the oncogene FOXP1 and PIK3IP1.
2015, Lee	T1+Flair	T, 65 pts	- 36 spatial habitat diversity (regions with distinctly different intensity characteristics) features based on pixel abundances w/in ROIs. - Overall coefficient of variation, symbolic regression method.	- Association with OS and EGFR+ status - Could be a useful prognostic tool for MRIs of patients with glioblastomas.
2016, Kickingereder	T1, T1+ Flair	T, 112 pts V, 60 pts	- 4842 total - 17 first-order features, 9 volume and shape features, 162 texture features. - Supervised principal component analysis, Cox proportional hazard models, integrated Brier scores.	- An 72-feature radiomics-based classification of recurrent glioblastoma permits the prediction of treatment outcome to antiangiogenic therapy through PFS and OS.
2016, Kickingereder	T1+ Flair	T, 79 pts V, 40 pts	- 12,190 indexes - Supervised principal component analysis.	- An 11-feature radiomic signature allowed prediction of PFS and OS, stratification of patients with newly diagnosed glioblastoma, and improved performance compared with that of established clinical and radiologic risk models.
2016, Grossmann	T1+ FLAIR	T, 144 pts (gene, 91 pts)	- Volumetric features such as the necrotic core, contrast enhancement, abnormal tumor volume, tumor-associated edema, and total tumor volume (TV), as well as ratios of these tumor components. - Spearman rho, C-index, Noether test.	- Association of imaging features with immune response pathways and apoptosis, signal transduction and protein folding processes, homeostasis and cell cycling pathways, as well as OS.
2016, McGarry	T1, T1+ ADC FLAIR	T, 81 pts	- Map containing 81 (3^4^) potential voxel-wise codes. A 4-digit code was assigned to each voxel. The digit order chosen was T1, ADC, T1+, and FLAIR. Codes ranged from 1111 (dark voxels on all images) to 3333. - Log-rank Kaplan-Meier survival analysis, Cox proportional hazards model, combined classifier.	- Radiomic signature predicted poorer prognosis at tumor diagnosis in newly diagnosed glioblastoma
2017, Prasanna	T1 FLAIR T2	T, 65 pts	- 402 radiomic features were obtained for each region: enhancing lesion, peritumoral brain zone and tumor necrosis. - Redundancy maximum relevance feature selection , random forest (RF) classifier, threefold cross-validation.	- Ten radiomic “peritumoral” MRI features, suggestive of intensity heterogeneity and textural patterns, were predictive of survival on treatment-naïve pre-operative glioblastoma.
2017, Yu	FLAIR	T, 110 pts V, 30 pts	- 671 high-throughput features were extracted from grade II glioma. - Classification by support vector machine and AdaBoost, leave-one-out cross-validation.	- 110 features were selected for the noninvasive IDH1 status estimation of grade II glioma.
2017, Xi	T1, T1+ T2	T, 98 pts V, 20 pts	- 1665 imaging features - Reduced using LASSO regularization, classification by support vector machine.	- The best classification system for predicting MGMT promoter methylation status in preoperative gliobastoma originated from the combination of 36 T1, T2, and enhanced T1 images features.
2017, Kickingereder	T1, T1+ FLAIR T2	T, 120 pts V, 60 pts	- 1043 imaging features - Penalized Cox model with 10-fold cross-validation.	- The 8-feature radiomic signature increased the prediction accuracy for PFS and OS beyond the assessed molecular, clinical, and standard imaging parameters in newly diagnosed glioblastoma prior to standard-of-care treatment.
2017, Li	T1+	T, 96 pts	- 555 imaging features - Student’s tests (*t* test)	- Glioblastoma in different age groups (< 45 and ≥ 45 years old) present different radiomics-feature patterns, suggesting different pathologic, protein, or genic origins. - 101 features showing the consistency with the age groups, and unsupervised clustering results of those features also show coherence with the age difference.
2017, Grossmann	T1+ FLAIR	T, 126 pts V, 165 pts	- 65 imaging features from T1 and FLAIR scans at baseline (pretreatment) and follow-up after 6 weeks (post treatment initiation) - Unbiased unsupervised feature selection (PCA), selection of variant features (coefficient of variation). - Minimal redundancy maximal relevance algorithm, Cox proportional hazards model for PFS or OS.	- Multivariable analysis of features derived at baseline imaging resulted in significant stratification of OS and PFS. - These stratifications were stronger compared with clinical or volumetric covariates prognostic value for survival and progression in patients with recurrent glioblastoma receiving bevacizumab treatment.
2017, Kanas	T1+ FLAIR	T, 86 pts	- 10 quantitative variables and 24 qualitative variables were calculated from the volumes of three distinct regions: edema/invasion, tumor enhancement (tumor), and necrosis. - Isometric feature mapping, locally linear embedding, Laplacian eigenmaps, linear discriminant analysis, factor analysis, principal components analysis, stochastic proximity embedding, random forest, k-nearest neighbors, Gaussian naive Bayes, and the J48 tree.	- The status of MGMT promoter methylation was predicted with an accuracy of up to 73.6%. - Experimental analysis showed that the edema/necrosis volume ratio, tumor/necrosis volume ratio, edema volume, and tumor location and enhancement characteristics were the most significant variables in respect to the status of MGMT promoter methylation in glioblastoma.
2010, Drabycz	T1+ T2 FLAIR	T, 59 pts	- 4 visual qualitative texture features (cysts, ring/nodular enhancement, margins, homogeneity), volume, 11 regions/sectors features and space–frequency texture analysis based on the S-transform. - Two-way repeated-measures analysis of variance (ANOVA) tests.	- Ring enhancement assessed visually is significantly associated with unmethylated MGMT promoter status. -Texture features on T2 images assessed by the space–frequency analysis were significantly different between methylated and unmethylated cases.

*Flas* fast low-angle shot, *OS* overall survival, *PFS* progression free survival, *MGMT* O6-methylguanine-DNA-methyltransferase, *IDH* isocitrate deshydrogenase

Machine-learning algorithms and AI signatures were trained to predict overall survival in patients with solid tumors treated with ICM based on pretreatment-imaging biomarkers. These biomarkers, predictors of poorer outcomes, can be macroscopic such as the presence of a higher tumor burden and sarcopenia [66] or microscopic such as an AI-signature estimating CD8 cell counts and predicting clinical outcomes of patients treated with immunotherapy [67]. One of the most promising fields is the evaluation of intrinsic glioblastoma heterogeneity, which is due to the coexistence of distinct sub-clones and also regional intrinsic plasticity shaped by tumor microenvironment [68]. In addition, it exists also an important inter-tumor heterogeneity with variable expression levels of surface biomarkers [69]. These phenotypic heterogeneities explain treatment resistances developed by glioblastoma. AI can be trained to decipher spatial and temporal glioblastoma heterogeneity which is a major driver of the poor prognosis of glioblastoma patients [70]. On a larger perspective, tumor heterogeneity evolution in space and time under immune selection is the major obstacle to personalized-medicine and biomarker development [71].

The use of AI in glioblastoma patients is primarily in the field of diagnosis and treatment plan. The vast majority of current studies (Table 4) used standard of care MRI sequences and combined several features extracted from both unenhanced and enhanced sequences [72]. The typical radiomics pipeline involved the delineation of the tumor on medical images, then the calculation of imaging features in this volume of interest (i.e., using mathematical formulas defined a priori or identified directly by the computer using deep-learning) and finally the creation of prognostic or predictive models using these features. AI identified several signatures associated with methylation [72, 73], age-related patterns [74] and prognosis factors [75, 76].

Few studies explored AI to guide treatment monitoring and follow-up: AI identified patterns associated with treatment response [77, 78] such as enhancement patterns in antiangiogenic therapies [77]. Since the majority (83%) of centers prefer to undertake qualitative assessments of response rather than using RANO criteria [4], AI could be used to standardize evaluations across institutions rather than relying on the interpretation of expert radiologists which is inherently subjective.

## Limitations of conventional non-immune-specific PET biomarkers

PET imaging is the procedure of choice for image-based quantification of biological processes (Table 3), as it provides at least three main advantages compared to MRI in this setting specifically: (1) its detection sensitivity is more than 10^3^ times higher, (2) the direct proportionality between the PET numerical signal and biological tracers’ concentration allows powerful image-based quantification, and (3) finally the possibility to combine any biological vector of interest to a radiomarker has virtually no limits. However, radiochemistry capabilities, availability, and cost of several radiotracers remain major limitations.

### Glucose metabolism

Increased glucose consumption is a hallmark of cancers [79], but the critical role of glycolysis in the function of many immune cells has also resulted in ^18^F-FDG PET being used to measure immune responses. Although increased ^18^F-FDG PET uptake is observed in high-grade tumors [80], poorer prognosis [81], and anaplastic transformation [82] (Fig. 3), the lack of specificity of glucose consumption [83] makes its applications uncertain for the assessment of gliobastoma response to ICM in monotherapy [84]. Moreover, recent researches have shown that FDG accumulates mostly in innate immune cells, and so ^18^F-FDG PET seems to be more useful in evaluating the effects of therapies that target inflammatory mediators than in monitoring cell expansion [85]. However, high FDG-uptake could be used in combination therapies to predict radiation therapy failure [86], antiangiogenic failure [87], and poorer outcome, as well as tumor recurrence [82].

### Amino acid metabolism

The growth of proliferating glioblastoma cells relies on a large neutral amino acid transport system. These amino acids are used as the natural building blocks of proteins and to detect high-grade tumors. The most frequently used radiolabeled amino acid are ^18^F-FET (fluor-18 Fluoro-ethyl-l-tyrosine) [88, 89], ^11^C-methionine, alpha-^11^C-l-methyltryptophan (AMT) [90], and ^18^F-FDOPA [91, 92]*.* The use of amino acids could provide a breakthrough in the evaluation of response to ICM therapies in monotherapy or combinations [93]. Indeed, amino acid uptake is independent of regional tumor perfusion and blood-brain barrier permeability, and the large neutral amino acid transport system is specifically overexpressed by tumor cells [89, 94] regardless of the breakup of the blood-brain barrier contrary to MRI and gadolinium-enhancement [95]. An early decrease in PET amino acid uptake outperformed MRI for early prediction of recurrence [88], outcome [96], and response to chemotherapy, bevacizumab, or VEGF inhibitor [97]. ^18^F-FET PET detected pseudoprogression in glioblastoma treated with bevacizumab [98], as well as in melanoma brain metastasis treated with ICMs [99]. The main limitation for response assessment in glioblastoma patients treated with ICMs is the lack of prospective data.

### DNA synthesis

An increased cellular proliferation rate is a hallmark of malignancy and requires DNA synthesis. Nucleoside analogs such as ^18^F-FLT (3′-(^18^F)-Fluoro-3′-deoxythymidine) [100] are phosphorylated and trapped in cells synthesizing DNA [101]. Consequently, ^18^F-FLT uptake is associated with a high signal to noise ratio (i.e., low uptake in normal brain tissue), and strongly correlated to cellular proliferation (i.e., Ki-67) in brain tumors [102]. The main advantage is the possibility of dynamic evaluation of the kinetics of the radiotracer biodistribution. The limitations are that FLT uptake in brain tissue requires a disruption of the blood-brain barrier and is increased by inflammation [101]. Clinical value in ICMs has not been evaluated although there is a rationale suggesting that ^18^F-FLT PET could be useful for response evaluation since it is a surrogate marker of angiogenesis and proliferation [52]. An early decrease in ^18^F-FLT uptake [103] predicted prolonged survival in patients treated with bevacizumab plus Irinotecan.

### Membrane proliferation

^18^F-fluorocholine is a widely available PET tracer that is a small precursor molecule for the synthesis of membrane phospholipids. ^18^F-fluorocholine PET can predict early response in glioblastoma treated with radiotherapy and temozolomide [104]. Clinical value in ICMs has not been evaluated. However, the limitation is that choline is increased in inflammatory processes (false positives) [105], and its brain uptake is strongly affected by disturbances of the blood-brain barrier observed in high-grade gliomas [106].

### Angiogenesis and perfusion

H2^15^O PET remains the reference standard for cerebral blood flow evaluation [107], however, its use is not possible without a cyclotron on site (half-life of ^15^O = 2 min), making its use not possible in clinical practice. Novel PET tracers are in development such as integrins that are glycoproteins involved in cell-to-matrix relationships[108], which can be evaluated by PET (^18^F-AlF-NOTA-PRGD2 PET, ^18^F FPPRGD2 PET). The literature about radiolabeled integrin is scarce. Integrins were used to diagnose glioblastoma and predicted early response to conventional treatment and bevacizumab failure [109] and therefore could be used to evaluate ICM in combination therapies.

### Hypoxia

Hypoxia promotes an immunosuppressive environment, therapy resistance, and disease recurrence [10, 110]. Hypoxia can be detected using specific PET radiotracers such as ^15^Oxygen, ^18^F-Fluoromisonidazole (^18^F-FMISO) [111], and ^18^F-1-(5-fluoro-5-deoxy-α-D-arabinofuranosyl)-2-nitroimidazole (^18^F-FAZA). The main limitation is that the signal to noise ratio of PET radiotracers targeting hypoxia is low compared to normal brain tissue. Increased hypoxia measured by ^18^F-MISO PET can be used in treatment planning since it predicted shorter survival and could be used in radiotherapy planning to boost treatment in hypoxic and potentially radio-resistant areas. ^18^F-FMISO could play a significant role in the monitoring of bevacizumab therapy [58], as well as in the monitoring of ICMs [59] since aberrant hypoxic neovascularity is associated with immunosuppressive environments.

### Mitochondrial activity

The mitochondrial translocator protein (TSPO) is overexpressed in activated microglia [112]. TSPO have been mostly developed to investigate neuroinflammatory processes. The main limitations are that they are not yet available in daily practice and there are nonoptimal imaging properties since its uptake in glioblastoma lesions is more likely to be due to simple breakdown of the blood-brain barrier. TSPO was only evaluated in diagnostic settings and was never evaluated for response assessment. ^18^F-GE-180 is a novel third generation TSPO receptor ligand with high binding affinity compared to existent radiotracers and with better diagnostic performances than MRI [113].

### Somatostatin receptors

Somatostatin receptor (SSTR) expression can be measured by scintigraphy or PET [114]. In the majority of glioblastomas, the expression of SSTR2 is negative (the most commonly expressed is SSTR5) [115]. Theoretically, the expression of SSTR2 by activated immune cells such as leukocytes and macrophages could be detected and used to characterize the inflammatory infiltrate in patients treated with ICM [116]. However, a limitation of this approach is that the disruption of the blood-brain barrier in high-grade gliomas may increase somatostatin receptor ligand uptake.

## Immune-specific PET biomarkers under investigation

### Rationale

Glioblastoma is a heterogeneous immunosuppressive microenvironment. While ICMs aim to restore tumor elimination by immune cells, to date imaging techniques used in clinical routine and in research have mainly focused on tumor cells rather than the immune environment. Nonetheless, the immune context, which is determined by the density, composition, functional state, and organization of the leukocyte infiltrate of the tumor, predicts the efficacy of ICMs. Although this immune contexture can be used to predict prognosis and treatment response and undergoes temporal changes in case of immune responses, it is not being evaluated by current clinical trials (Table 1).

In the future, the strategy could shift to substitute non-immune-specific imaging biomarkers by immune-specific biomarkers. Innovations in chemistry allowed to produce radiotracers targeting PD-1 or PD-L1 (lymphocytic exhaustion) [117], CD8 (cytotoxic lymphocytes) [118], or IL2 (activated lymphocytes) [119]. This whole body in vivo assessment of the density of receptors and ligands involved in lymphocyte activation might provide more comprehensive information than ex vivo immunohistochemistry provided by single biopsy samples. There are indeed various publications showing the promising results of immuno-PET [120] using antibodies, diabodies, or small molecules (Table 1).

### Radiolabeled ICM: PD-(L)1

There is a strong rationale suggesting that the promising group of radiotracers targeting PD-1 or its ligand (PD-(L)1) will be increasingly used. First, PD-(L)1 PET could guide treatment planning. Although these radiotracers do not discriminate PD-L1 expression on tumor cells and immune cells, PET can quantify non-invasively tumor heterogeneity. As a comparison, the current reference standard is immunohistochemistry which allows evaluating PD-L1 expression on tumor cells and immune cells on a biopsy sample. However, immunohistochemistry is an invasive technique, which is limited by the temporal and spatial heterogeneity of glioblastoma’s PD-L1 expression [121]. Second, PD-(L)1 PET could be used to monitor and predict ICM efficacy. In animal models, an effective immunoradiotherapy increases the expression of PD-1 and tumor infiltration by PD-1+ lymphocytes [122]. Finally, these radiotracers could be used to evaluate in vivo the pharmacokinetics and biodistribution of ICM.

PD-(L)1 imaging is being investigated prospectively in several clinical trials in melanoma, NSCLC, breast, and bladder cancers but not in patients treated with glioblastoma. Current radiotracers include the high-affinity engineered protein scaffold (HAC-PD-1) that can detect human PD-L1 expression 1 h after injection [123], anti-PD-L1 antibodies [122, 124, 125], anti-PD-1 antibodies [122, 126], and small non-antibodies PD-L1-specific peptides [127, 128]. The radiolabeling of these agents used either positron emitters or single-photon emitters such as ^64^Cu [122], ^89^Zr [125], ^18^F [128], ^111^In [124], and ^99^mTc [127].

There are currently two different strategies for PD-(L)1 imaging. On the one hand, anti-PD-(L)1 antibodies can accumulate in tissue but suffer from lower tumor penetration, long retention in the blood pool, and poor signal to noise ratio. Additionally, higher doses need to be injected and imaging must be performed several days after injection [117, 122]. On the other hand, non-antibody small molecules with high affinity for PD-L1 allow an efficient penetration in to the tumor, as well as high signal-to-noise ratios. Imaging can therefore be performed within a few hours after injection and requires lower activities, and there is a faster clearance by the kidneys [127].

### CD8 imaging

Cluster of differentiation 8 (CD8) is a transmembrane glycoprotein and a co-receptor for the T cell receptor (TCR), which is specific to class I MHC proteins. MHC class I displays fragments of non-self-peptides derived from cytosolic proteins, which will trigger an immediate response from the immune system against tumor cells [129]. There is a strong rationale demonstrating that high intratumor CD8 expression is associated with better outcome and could be used to predict or monitor ICM treatment efficiency.

Radiolabeled PET agents have been developed to target and identify CD8 in vivo [130] but are not yet used in human research. Alternatively*,* MRI imaging of CD8+ T-Lymphocytes recruitment was investigated in an experimental mice model. CD8+ T-lymphocytes labeled with superparamagnetic iron oxide accumulated in the tumor 24 h after injection [131]. The limitation of MRI tracking is the quantity of superparamagnetic necessary to obtain a good signal while PET radiotracers require nonpharmacologic doses.

### Tumor-associated macrophages imaging

Tumor-associated-macrophages (TAMs) are major components of glioblastoma microenvironment and overexpress the immunosuppressive PD-1 ligand [132, 133]. There are two subpopulations and two phenotypes of TAMs. The subpopulations include microglia and monocyte-derived macrophages[133], and two phenotypes have been described: M1 and M2. An increased number of TAMs with a M2-like phenotype is associated with a poorer prognosis [134] and promotes tumor angiogenesis and immune-suppression [135]. Imaging biomarkers targeting specifically TAMs could be leveraged to guide precision approaches in patients treated with ICM in monotherapy or in combination since macrophages are becoming an increasingly important target for cancer therapy.

Several strategies were developed to detect the presence of TAMs in vivo [136]*.* First, activated macrophages are extremely FDG avid and can be detected by FDG PET, but there is a need for more specific biomarkers in glioblastoma patients [83]. Second, CD206 is a receptor overexpressed on M2 macrophages which can be detected through single-photon emission computed tomography (SPECT) imaging (^99m^Tc-labeled anti-CD206 and ^125^I-αCD206) and optical imaging (Dye-αCD206) [135]. SPECT and infrared fluorescence imaging using an anti-CD206 monoclonal antibody were used as early biomarkers to predict post-chemotherapy tumor relapse [135]. Third, gadolinium tagged with a fluorescent poly (l-glutamic acid) was used to detect TAMs in rat glioma model since it is co-localized with CD68 (a marker for macrophages) and CD169 (marker for activated macrophages) [137].

### Interleukin-2 imaging

Activated T lymphocytes, especially CD4+ and CD8+ Th1 (T helper) lymphocytes, produce Interleukin-2 (IL-2). This cytokine produced after antigen stimulation plays pivotal and complex roles in both the immune response and limiting inappropriate immune reactions. IL-2 mediates diverse pleiotropic actions, promoting T cell proliferation, survival, cytolytic activity, NK cell activity, development of regulatory T cells, and activation-induced cell death [138]. Because IL-2 is a cornerstone in the immune environment, radiolabeled agents are developed to target and identify interleukin 2 in vivo [119]. These new biomarkers could be useful in ICMs.

### Other biomarkers of inflammation

Many PET radiotracers are available to characterize specific components of the inflammatory process [139]: neovascularization (18F-RGD targeting α_v_β_3_), Cyclooxygenase (^11^C-celecoxib), matrix metalloproteinase (^18^F-CGS27023A), microglia (^11^C-GW405833 targeting CB_2_R, ^64^Cu-DOTA-etanercept targeting TNFR, ^18^F-DPA-714 targeting TSPO), neutrophils (^64^Cu-PEG-cFLFLFK targeting FPR, ^18^F-FDG transported by glut), B cells (^124^I-rituximab targeting CD20, ^18^F-FDG transported by glut), T cells (^18^F-FB-IL2 targeting IL2R, ^18^F-FDG transported by glut), and macrophages (^68^Ga-DOTA-TOC targeting SSTR, ^18^F-FDG transported by glut, ^64^Cu-DOTA-etanercept targeting TNFR, ^18^F-RGD targeting α_v_β_3_, ^18^F-DPA-714 targeting TSPO). These radiotracers seem promising for detecting the inflammatory process and could be used to decipher immune contexture or identify pseudoprogression.

### Conclusion and perspectives

This review summarizes perspectives on the emerging trends in medical imaging for optimizing treatments in glioblastoma patients treated with anti-CTLA4 and anti-PD-1 agents in monotherapy or in combination, as well as on the potential biomarkers that might improve the early identification of patients that will benefit from those treatments.

Evaluating the efficacy of ICMs is challenging because it triggers new radiological patterns of response and progression such as hyperprogression, pseudoprogression, abscopal effect, and immune-related adverse events. Immunotherapy response assessment for neuro-oncology (iRANO) criteria [3], define a “wait and see strategy” for progressive patients treated with ICMs in monotherapy. However, a recent survey demonstrated that a minority of centers use RANO criteria [4], and we observed that a minority of clinical trials implemented iRANO criteria (Table 1). This lack of quantitative assessment demonstrates the need for standardized evaluation and the development of quantitative algorithms for robust response assessments.

Our review listed studies using MRI and PET techniques and demonstrates that there is a lot of noise in the current heterogeneous literature. Our insight and impression is that future prospective clinical work is still needed and that the most promising imaging modalities are standard of care MRI, aminoacid PET, and immunoPET. Additionally, the major concrete recommendation from our review is that the optimal imaging modality related to these imaging challenges in clinical routine remains MRI since it is the only technique with sufficient clinical evidences and with specific immune-related evaluation criteria (iRANO). The limitation of all advanced MRI techniques is indeed the lack of standardization and robustness combined with a disease where biopsy confirmation is difficult and biased, making it very difficult to recommend other options than further studies are recommended. A main limitation for PET tracers is transport across the blood-brain barrier. This has limited to permanently establish them for clinical use since the tumor is simply not detected with sufficient sensitivity. This includes most of the tracers mentioned. Nonetheless, specific tracers such as amino acid tracers have potential value since amino acid transport is independent from the intact or disrupted blood-brain barrier. The level of evidence of data presented in the literature remains speculative. All these points need to be clarified by future researches.

The most promising field is the use of new bioengineering techniques, which allow the targeting of probes deciphering the immune contexture, while datamining techniques and artificial intelligence will fully exploit and quantify the existing information from conventional imaging techniques. Further development of the new concept of supervoxels could capitalize and combine these two approaches, thereby redefining medical imaging as a comprehensive and quantitative decision tool characterizing the tumor and its environment. Artificial intelligence could excel in combining all this information and extract synthetic quantitative probability guiding the decision to start, continue or stop ICM in monotherapy or combination.

## Data Availability

NA.

## Abbreviations

^18^F-FDOPA: ^18^F-fluorodopamine
^18^F-FET: ^18^F-Fluoro-ethyl-l-tyrosine
^18^F-FLT: 3′-(^18^F)-Fluoro-3′-deoxythymidine
^18^F-FMISO: ^18^F-Fluoromisonidazole
ADC: Apparent diffusion coefficient
BBB: Blood-brain barrier
BOLD: Blood-oxygen level dependent
CBF: Cerebral blood flow
CBV: Cerebral blood volume
Cho: Choline
Cr: Creatinine
CTLA-4: Cytotoxic T-lymphocyte antigen-4
DCE: Dynamic contrast-enhanced
DNA: Deoxyribonucleic acid
EGF: Epithelial growth factor
FDG: Fluorodeoxyglucose
ICM: Immune checkpoint modulators
IDH: Isocitrate dehydrogenase
IL: Interleukin
MGMT: O6-methylguanine-DNA-methyltransferase
MHC: Major histocompatibility complex
MRI: Magnetic resonance imaging
MRSI: Magnetic resonance spectroscopic imaging
NAA: *N*-Acetyl-Aspartate
PD-1: Programmed death 1
PD-L1: Programmed death-ligand 1
PET: Positron-emission tomography
RANO: Response assessment for neuro-oncology
TAMs: Tumor-associated-macrophages
TSPO: Translocator protein
VEGF: Vascular endothelial growth factor

## References

[1] Stupp R, Hegi ME, Mason WP, van den Bent MJ, Taphoorn MJB, Janzer RC (2009). Effects of radiotherapy with concomitant and adjuvant temozolomide versus radiotherapy alone on survival in glioblastoma in a randomised phase III study: 5-year analysis of the EORTC-NCIC trial. Lancet Oncol..

[2] Pardoll DM (2012). The blockade of immune checkpoints in cancer immunotherapy. Nat Rev Cancer..

[3] Okada H, Weller M, Huang R, Finocchiaro G, Gilbert MR, Wick W (2015). Immunotherapy response assessment in neuro-oncology: a report of the RANO working group. Lancet Oncol..

[4] Thust SC, Heiland S, Falini A, Jäger HR, Waldman AD, Sundgren PC (2018). Glioma imaging in Europe: a survey of 220 centres and recommendations for best clinical practice. Eur Radiol..

[5] Tighe Joseph, Lazebnik Svetlana (2010). SuperParsing: Scalable Nonparametric Image Parsing with Superpixels. Computer Vision – ECCV 2010.

[6] Dhermain FG, Hau P, Lanfermann H, Jacobs AH, van den Bent MJ (2010). Advanced MRI and PET imaging for assessment of treatment response in patients with gliomas. Lancet Neurol..

[7] Shah JL, Li G, Shaffer JL, Azoulay MI, Gibbs IC, Nagpal S (2018). Stereotactic radiosurgery and hypofractionated radiotherapy for glioblastoma. Neurosurgery..

[8] Biau J, Chautard E, De Schlichting E, Dupic G, Pereira B, Fogli A (2017). Radiotherapy plus temozolomide in elderly patients with glioblastoma: a “real-life” report. Radiation Oncology..

[9] Castro BA, Aghi MK (2014). Bevacizumab for glioblastoma: current indications, surgical implications, and future directions. Neurosurg Focus..

[10] Corroyer-Dulmont A, Peres EA, Gerault AN, Savina A, Bouquet F, Divoux D (2016). Multimodal imaging based on MRI and PET reveals [ ^18^F]FLT PET as a specific and early indicator of treatment efficacy in a preclinical model of recurrent glioblastoma. European Journal of Nuclear Medicine and Molecular Imaging..

[11] Wen PY, Kesari S (2008). Malignant gliomas in adults. N Engl J Med..

[12] Crocetti E, Trama A, Stiller C, Caldarella A, Soffietti R, Jaal J (2012). Epidemiology of glial and non-glial brain tumours in Europe. Eur J Cancer..

[13] Thakkar JP, Dolecek TA, Horbinski C, Ostrom QT, Lightner DD, Barnholtz-Sloan JS (2014). Epidemiologic and molecular prognostic teview of glioblastoma. Cancer Epidemiol Biomarkers Prev..

[14] Lamborn KR, Chang SM, Prados MD (2004). Prognostic factors for survival of patients with glioblastoma: recursive partitioning analysis. Neuro-oncol..

[15] Driessens G, Kline J, Gajewski TF (2009). Costimulatory and coinhibitory receptors in anti-tumor immunity. Immunol Rev..

[16] Kamran N, Calinescu A, Candolfi M, Chandran M, Mineharu Y, Asad AS (2016). Recent advances and future of immunotherapy for glioblastoma. Expert Opin Biol Ther..

[17] Jackson CM, Lim M, Drake CG (2014). Immunotherapy for brain cancer: recent progress and future promise. Clin Cancer Res..

[18] Preusser M, Lim M, Hafler DA, Reardon DA, Sampson JH (2015). Prospects of immune checkpoint modulators in the treatment of glioblastoma. Nat Rev Neurol..

[19] Ghosh D, Nandi S, Bhattacharjee S (2018). Combination therapy to checkmate glioblastoma: clinical challenges and advances. Clin Transl Med..

[20] Derer A, Spiljar M, Bäumler M, Hecht M, Fietkau R, Frey B, et al. Chemoradiation increases PD-L1 expression in certain melanoma and glioblastoma cells. Front Immunol [Internet]. 2016 [cited 2018 Oct 19];7. Available from: https://www.ncbi.nlm.nih.gov/pmc/articles/PMC5177615/10.3389/fimmu.2016.00610 PMC5177615 28066420

[21] Burnette B, Weichselbaum RR (2013). Radiation as an immune modulator. Semin Radiat Oncol..

[22] Suwa T, Saio M, Umemura N, Yamashita T, Toida M, Shibata T (2006). Preoperative radiotherapy contributes to induction of proliferative activity of CD8+ tumor-infiltrating T-cells in oral squamous cell carcinoma. Oncol Rep..

[23] Norden AD, Young GS, Setayesh K, Muzikansky A, Klufas R, Ross GL (2008). Bevacizumab for recurrent malignant gliomas: efficacy, toxicity, and patterns of recurrence. Neurology..

[24] Gilbert MR, Dignam JJ, Armstrong TS, Wefel JS, Blumenthal DT, Vogelbaum MA (2014). A randomized trial of bevacizumab for newly diagnosed glioblastoma. New England Journal of Medicine..

[25] Terme M, Colussi O, Marcheteau E, Tanchot C, Tartour E, Taieb J (2012). Modulation of immunity by antiangiogenic molecules in cancer. Clin Dev Immunol..

[26] Dercle L, Seban R-D, Lazarovici J, Schwartz LH, Houot R, Ammari S (2018). 18F-FDG PET and CT scans detect new imaging patterns of response and progression in patients with Hodgkin lymphoma treated by Anti-Programmed Death 1 Immune Checkpoint Inhibitor. J Nucl Med..

[27] Beer L, Hochmair M, Prosch H (2018). Pitfalls in the radiological response assessment of immunotherapy. Memo..

[28] Wen PY, Macdonald DR, Reardon DA, Cloughesy TF, Sorensen AG, Galanis E (2010). Updated response assessment criteria for high-grade gliomas: response assessment in neuro-oncology working group. J Clin Oncol..

[29] Balaña C, Capellades J, Pineda E, Estival A, Puig J, Domenech S (2017). Pseudoprogression as an adverse event of glioblastoma therapy. Cancer Med..

[30] Champiat S, Dercle L, Ammari S, Massard C, Hollebecque A, Postel-Vinay S (2017). Hyperprogressive disease is a new pattern of progression in cancer patients treated by Anti-PD-1/PD-L1. Clin Cancer Res..

[31] Fuentes-Antrás J, Provencio M, Díaz-Rubio E (2018). Hyperprogression as a distinct outcome after immunotherapy. Cancer Treatment Reviews..

[32] Michot J-M, Mazeron R, Dercle L, Ammari S, Canova C, Marabelle A (2016). Abscopal effect in a Hodgkin lymphoma patient treated by an anti-programmed death 1 antibody. Eur J Cancer..

[33] Chinot OL, Macdonald DR, Abrey LE, Zahlmann G, Kerloëguen Y, Cloughesy TF (2013). Response assessment criteria for glioblastoma: practical adaptation and implementation in clinical trials of antiangiogenic therapy. Curr Neurol Neurosci Rep..

[34] Mekki A, Dercle L, Lichtenstein P, Marabelle A, Michot J-M, Lambotte O (2018). Detection of immune-related adverse events by medical imaging in patients treated with anti-programmed cell death 1. Eur J Cancer..

[35] Albert NL, Weller M, Suchorska B, Galldiks N, Soffietti R, Kim MM (2016). Response Assessment in Neuro-Oncology working group and European Association for Neuro-Oncology recommendations for the clinical use of PET imaging in gliomas. Neuro Oncol..

[36] Valentini MC, Mellai M, Annovazzi L, Melcarne A, Denysenko T, Cassoni P (2017). Comparison among conventional and advanced MRI, 18F-FDG PET/CT, phenotype and genotype in glioblastoma. Oncotarget..

[37] Fudaba H, Shimomura T, Abe T, Matsuta H, Momii Y, Sugita K (2014). Comparison of multiple parameters obtained on 3T pulsed arterial spin-labeling, diffusion tensor imaging, and MRS and the Ki-67 labeling index in evaluating glioma grading. American Journal of Neuroradiology..

[38] Paldino MJ, Desjardins A, Friedman HS, Vredenburgh JJ, Barboriak DP (2012). A change in the apparent diffusion coefficient after treatment with bevacizumab is associated with decreased survival in patients with recurrent glioblastoma multiforme. Br J Radiol..

[39] Ellingson BM, Gerstner ER, Smits M, Huang RY, Colen R, Abrey LE (2017). Diffusion MRI phenotypes predict overall survival benefit from Anti-VEGF monotherapy in recurrent glioblastoma: converging evidence from phase II trials. Clin Cancer Res..

[40] Qin L, Li X, Stroiney A, Qu J, Helgager J, Reardon DA (2017). Advanced MRI assessment to predict benefit of anti-programmed cell death 1 protein immunotherapy response in patients with recurrent glioblastoma. Neuroradiology..

[41] Vrabec M, Van Cauter S, Himmelreich U, Van Gool SW, Sunaert S, De Vleeschouwer S (2011). MR perfusion and diffusion imaging in the follow-up of recurrent glioblastoma treated with dendritic cell immunotherapy: a pilot study. Neuroradiology..

[42] Reimer C, Deike K, Graf M, Reimer P, Wiestler B, Floca RO, et al. Differentiation of pseudoprogression and real progression in glioblastoma using ADC parametric response maps. PLoS One [Internet]. 2017 [cited 2018 Oct 19];12. Available from: https://www.ncbi.nlm.nih.gov/pmc/articles/PMC5383222/10.1371/journal.pone.0174620 PMC5383222 28384170

[43] Auer TA, Breit H-C, Marini F, Renovanz M, Brockmann MA, Tanyildizi Y. Evaluation of the apparent diffusion coefficient in patients with recurrent glioblastoma under treatment with bevacizumab with radiographic pseudoresponse. J Neuroradiol. 2018. 10.1016/j.neurad.2018.04.002 29733920

[44] Hilario A, Ramos A, Perez-Nuñez A, Salvador E, Millan JM, Lagares A (2012). The added value of apparent diffusion coefficient to cerebral blood volume in the preoperative grading of diffuse gliomas. AJNR Am J Neuroradiol..

[45] Zakaria R, Platt-Higgins A, Rathi N, Radon M, Das S, Das K (2018). T-cell densities in brain metastases are associated with patient survival times and diffusion tensor MRI changes. Cancer Res..

[46] Bilgili Y, Unal B (2004). Effect of region of interest on interobserver variance in apparent diffusion coefficient measures. American Journal of Neuroradiology..

[47] Floeth FW, Wittsack HJ, Engelbrecht V, Weber F (2002). Comparative follow-up of enhancement phenomena with MRI and proton MR spectroscopic imaging after intralesional immunotherapy in glioblastoma--report of two exceptional cases. Zentralbl Neurochir..

[48] Smith MM, Thompson JE, Castillo M, Cush S, Mukherji SK, Miller CH (1996). MR of recurrent high-grade astrocytomas after intralesional immunotherapy. AJNR Am J Neuroradiol..

[49] Pallud J, Capelle L, Taillandier L, Fontaine D, Mandonnet E, Guillevin R (2009). Prognostic significance of imaging contrast enhancement for WHO grade II gliomas. Neuro Oncol..

[50] Liu Z-C, Yan L-F, Hu Y-C, Sun Y-Z, Tian Q, Nan H-Y (2017). Combination of IVIM-DWI and 3D-ASL for differentiating true progression from pseudoprogression of Glioblastoma multiforme after concurrent chemoradiotherapy: study protocol of a prospective diagnostic trial. BMC Med Imaging..

[51] Khalek Abdel Razek AA, El-Serougy L, Abdelsalam M, Gaballa G, Talaat M. Differentiation of primary central nervous system lymphoma from glioblastoma: Quantitative analysis using arterial-spin labeling and diffusion tensor imaging. World Neurosurg. 2018. 10.1016/j.wneu.2018.11.155 30502475

[52] Viel T, Boehm-Sturm P, Rapic S, Monfared P, Neumaier B, Hoehn M (2013). Non-invasive imaging of glioma vessel size and densities in correlation with tumour cell proliferation by small animal PET and MRI. Eur J Nucl Med Mol Imaging..

[53] Yun TJ, Cho HR, Choi SH, Kim H, Won J-K, Park S-W (2016). Antiangiogenic effect of bevacizumab: application of arterial spin-labeling perfusion MR imaging in a rat glioblastoma model. American Journal of Neuroradiology..

[54] Batchelor TT, Sorensen AG, di Tomaso E, Zhang W-T, Duda DG, Cohen KS (2007). AZD2171, a pan-VEGF receptor tyrosine kinase inhibitor, normalizes tumor vasculature and alleviates edema in glioblastoma patients. Cancer Cell..

[55] Hu LS, Eschbacher JM, Heiserman JE, Dueck AC, Shapiro WR, Liu S (2012). Reevaluating the imaging definition of tumor progression: perfusion MRI quantifies recurrent glioblastoma tumor fraction, pseudoprogression, and radiation necrosis to predict survival. Neuro-oncology..

[56] Corroyer-Dulmont A, Chakhoyan A, Collet S, Durand L, MacKenzie ET, Petit E, et al. Imaging modalities to assess oxygen status in glioblastoma. Front Med (Lausanne) [Internet]. 2015 [cited 2018 Aug 6];2. Available from: https://www.ncbi.nlm.nih.gov/pmc/articles/PMC4541402/10.3389/fmed.2015.00057 PMC4541402 26347870

[57] Hou BL, Bradbury M, Peck KK, Petrovich NM, Gutin PH, Holodny AI (2006). Effect of brain tumor neovasculature defined by rCBV on BOLD fMRI activation volume in the primary motor cortex. Neuroimage..

[58] Barajas RF, Krohn KA, Link JM, Hawkins RA, Clarke JL, Pampaloni MH, et al. Glioma FMISO PET/MR imaging concurrent with antiangiogenic therapy: molecular imaging as a clinical tool in the burgeoning era of personalized medicine. Biomedicines [Internet]. 2016 [cited 2018 Aug 6];4. Available from: https://www.ncbi.nlm.nih.gov/pmc/articles/PMC5344267/10.3390/biomedicines4040024 PMC5344267 28536391

[59] Spence AM, Muzi M, Swanson KR, O’Sullivan F, Rockhill JK, Rajendran JG (2008). Regional hypoxia in glioblastoma multiforme quantified with [18F]fluoromisonidazole positron emission tomography before radiotherapy: correlation with time to progression and survival. Clin Cancer Res..

[60] Gahramanov S, Muldoon LL, Varallyay CG, Li X, Kraemer DF, Fu R (2013). Pseudoprogression of glioblastoma after chemo- and radiation therapy: diagnosis by using dynamic susceptibility-weighted contrast-enhanced perfusion MR imaging with ferumoxytol versus gadoteridol and correlation with survival. Radiology..

[61] Gahramanov S, Varallyay C, Tyson RM, Lacy C, Fu R, Netto JP (2014). Diagnosis of pseudoprogression using MRI perfusion in patients with glioblastoma multiforme may predict improved survival. CNS Oncol..

[62] Toth GB, Varallyay CG, Horvath A, Bashir MR, Choyke PL, Daldrup-Link HE (2017). Current and potential imaging applications of ferumoxytol for magnetic resonance imaging. Kidney Int..

[63] Ma D, Gulani V, Seiberlich N, Liu K, Sunshine JL, Duerk JL (2013). Magnetic resonance fingerprinting. Nature..

[64] Gu Y, Wang CY, Anderson CE, Liu Y, Hu H, Johansen ML, et al. Fast magnetic resonance fingerprinting for dynamic contrast-enhanced studies in mice. Magn Reson Med. 2018. 10.1002/mrm.27345 PMC6226386 29744935

[65] Limkin EJ, Sun R, Dercle L, Zacharaki EI, Robert C, Reuzé S (2017). Promises and challenges for the implementation of computational medical imaging (radiomics) in oncology. Ann Oncol..

[66] Dercle L, Ammari S, Champiat S, Massard C, Ferté C, Taihi L (2016). Rapid and objective CT scan prognostic scoring identifies metastatic patients with long-term clinical benefit on anti-PD-1/-L1 therapy. Eur J Cancer..

[67] Sun R, Limkin EJ, Vakalopoulou M, Dercle L, Champiat S, Han SR (2018). A radiomics approach to assess tumour-infiltrating CD8 cells and response to anti-PD-1 or anti-PD-L1 immunotherapy: an imaging biomarker, retrospective multicohort study. Lancet Oncol..

[68] Prasanna P, Patel J, Partovi S, Madabhushi A, Tiwari P (2017). Radiomic features from the peritumoral brain parenchyma on treatment-naïve multi-parametric MR imaging predict long versus short-term survival in glioblastoma multiforme: Preliminary findings. Eur Radiol..

[69] Dirkse A, Golebiewska A, Buder T, Nazarov PV, Muller A, Poovathingal S (2019). Stem cell-associated heterogeneity in Glioblastoma results from intrinsic tumor plasticity shaped by the microenvironment. Nat Commun..

[70] Pirotte B, Goldman S, Massager N, David P, Wikler D, Vandesteene A (2004). Comparison of 18F-FDG and 11C-methionine for PET-guided stereotactic brain biopsy of gliomas. J Nucl Med..

[71] Angelova M, Mlecnik B, Vasaturo A, Bindea G, Fredriksen T, Lafontaine L (2018). Evolution of metastases in space and time under immune selection. Cell.

[72] Xi Y-B, Guo F, Xu Z-L, Li C, Wei W, Tian P (2018). Radiomics signature: a potential biomarker for the prediction of MGMT promoter methylation in glioblastoma. J Magn Reson Imaging..

[73] Kanas VG, Zacharaki EI, Thomas GA, Zinn PO, Megalooikonomou V, Colen RR (2017). Learning MRI-based classification models for MGMT methylation status prediction in glioblastoma. Comput Methods Programs Biomed..

[74] Li Z, Wang Y, Yu J, Guo Y, Zhang Q (2017). Age groups related glioblastoma study based on radiomics approach. Comput Assist Surg (Abingdon).

[75] Grossmann P, Narayan V, Chang K, Rahman R, Abrey L, Reardon DA (2017). Quantitative imaging biomarkers for risk stratification of patients with recurrent glioblastoma treated with bevacizumab. Neuro-oncology..

[76] McGarry SD, Hurrell SL, Kaczmarowski AL, Cochran EJ, Connelly J, Rand SD (2016). Magnetic resonance imaging-based radiomic profiles predict patient prognosis in newly diagnosed glioblastoma before therapy. Tomography..

[77] Diehn M, Nardini C, Wang DS, McGovern S, Jayaraman M, Liang Y (2008). Identification of noninvasive imaging surrogates for brain tumor gene-expression modules. Proc Natl Acad Sci USA..

[78] Rahman R, Hamdan A, Zweifler R, Jiang H, Norden AD, Reardon DA (2014). Histogram analysis of apparent diffusion coefficient within enhancing and nonenhancing tumor volumes in recurrent glioblastoma patients treated with bevacizumab. J Neurooncol..

[79] Boellaard R, Delgado-Bolton R, Oyen WJG, Giammarile F, Tatsch K, Eschner W (2015). FDG PET/CT: EANM procedure guidelines for tumour imaging: version 2.0. Eur J Nucl Med Mol Imaging.

[80] Manabe O, Hattori N, Yamaguchi S, Hirata K, Kobayashi K, Terasaka S (2015). Oligodendroglial component complicates the prediction of tumour grading with metabolic imaging. Eur J Nucl Med Mol Imaging..

[81] Herholz K, Langen K-J, Schiepers C, Mountz JM (2012). Brain tumors. Semin Nucl Med..

[82] Padma MV, Said S, Jacobs M, Hwang DR, Dunigan K, Satter M (2003). Prediction of pathology and survival by FDG PET in gliomas. J Neurooncol..

[83] Dercle L, Chisin R, Ammari S, Gillebert Q, Ouali M, Jaudet C (2015). Nonsurgical giant cell tumour of the tendon sheath or of the diffuse type: are MRI or 18F-FDG PET/CT able to provide an accurate prediction of long-term outcome?. Eur J Nucl Med Mol Imaging..

[84] Hodi FS, Butler M, Oble DA, Seiden MV, Haluska FG, Kruse A (2008). Immunologic and clinical effects of antibody blockade of cytotoxic T lymphocyte-associated antigen 4 in previously vaccinated cancer patients. Proc Natl Acad Sci USA..

[85] Nair-Gill E, Wiltzius SM, Wei XX, Cheng D, Riedinger M, Radu CG (2010). PET probes for distinct metabolic pathways have different cell specificities during immune responses in mice. J Clin Invest..

[86] Langleben DD, Segall GM (2000). PET in differentiation of recurrent brain tumor from radiation injury. J Nucl Med..

[87] Colavolpe C, Chinot O, Metellus P, Mancini J, Barrie M, Bequet-Boucard C (2012). FDG-PET predicts survival in recurrent high-grade gliomas treated with bevacizumab and irinotecan. Neuro-oncology..

[88] Pöpperl G, Götz C, Rachinger W, Gildehaus F-J, Tonn J-C, Tatsch K (2004). Value of O-(2-[18F]fluoroethyl)- L-tyrosine PET for the diagnosis of recurrent glioma. Eur J Nucl Med Mol Imaging..

[89] Rau FC, Weber WA, Wester H-J, Herz M, Becker I, Krüger A (2002). O-(2-[(18)F]Fluoroethyl)- L-tyrosine (FET): a tracer for differentiation of tumour from inflammation in murine lymph nodes. Eur J Nucl Med Mol Imaging..

[90] Bosnyák E, Michelhaugh SK, Klinger NV, Kamson DO, Barger GR, Mittal S (2017). Prognostic molecular and imaging biomarkers in primary glioblastoma. Clin Nucl Med..

[91] Bergmann R, Pietzsch J, Fuechtner F, Pawelke B, Beuthien-Baumann B, Johannsen B (2004). 3-O-methyl-6-18F-fluoro-L-dopa, a new tumor imaging agent: investigation of transport mechanism in vitro. J Nucl Med..

[92] Langen K-J, Hamacher K, Weckesser M, Floeth F, Stoffels G, Bauer D (2006). O-(2-[18F]fluoroethyl)-L-tyrosine: uptake mechanisms and clinical applications. Nucl Med Biol..

[93] Geier EG, Schlessinger A, Fan H, Gable JE, Irwin JJ, Sali A (2013). Structure-based ligand discovery for the Large-neutral Amino Acid Transporter 1, LAT-1. Proc Natl Acad Sci USA..

[94] Okubo S, Zhen H-N, Kawai N, Nishiyama Y, Haba R, Tamiya T (2010). Correlation of L-methyl-11C-methionine (MET) uptake with L-type amino acid transporter 1 in human gliomas. J Neurooncol..

[95] Upadhyay N, Waldman AD (2011). Conventional MRI evaluation of gliomas. Br J Radiol..

[96] Schwarzenberg J, Czernin J, Cloughesy TF, Ellingson BM, Pope WB, Geist C (2012). 3′-deoxy-3′-18F-fluorothymidine PET and MRI for early survival predictions in patients with recurrent malignant glioma treated with bevacizumab. J Nucl Med..

[97] Nedergaard MK, Michaelsen SR, Perryman L, Erler J, Poulsen HS, Stockhausen M-T (2016). Comparison of (18)F-FET and (18)F-FLT small animal PET for the assessment of anti-VEGF treatment response in an orthotopic model of glioblastoma. Nucl Med Biol..

[98] Galldiks N, Filss CP, Goldbrunner R, Langen K-J (2012). Discrepant MR and [(18)F]Fluoroethyl-L-Tyrosine PET imaging findings in a patient with bevacizumab failure. Case Rep Oncol..

[99] Kebir S, Rauschenbach L, Galldiks N, Schlaak M, Hattingen E, Landsberg J (2016). Dynamic O-(2-[18F]fluoroethyl)-L-tyrosine PET imaging for the detection of checkpoint inhibitor-related pseudoprogression in melanoma brain metastases. Neuro-oncology..

[100] Bollineni VR, Kramer GM, Jansma EP, Liu Y, Oyen WJG (2016). A systematic review on [(18)F]FLT-PET uptake as a measure of treatment response in cancer patients. Eur J Cancer..

[101] Shinomiya A, Miyake K, Okada M, Nakamura T, Kawai N, Kushida Y (2013). 3′-Deoxy-3′-[(18)F]-fluorothymidine ([(18)F]-FLT) transport in newly diagnosed glioma: correlation with nucleoside transporter expression, vascularization, and blood-brain barrier permeability. Brain Tumor Pathol..

[102] Chalkidou A, Landau DB, Odell EW, Cornelius VR, O’Doherty MJ, Marsden PK (2012). Correlation between Ki-67 immunohistochemistry and 18F-fluorothymidine uptake in patients with cancer: a systematic review and meta-analysis. Eur J Cancer..

[103] Wardak M, Schiepers C, Cloughesy TF, Dahlbom M, Phelps ME, Huang S-C (2014). 18F-FLT and 18F-FDOPA PET kinetics in recurrent brain tumors. Eur J Nucl Med Mol Imaging..

[104] Bolcaen J, Acou M, Boterberg T, Vanhove C, De Vos F, Van den Broecke C (2017). 18F-FCho PET and MRI for the prediction of response in glioblastoma patients according to the RANO criteria. Nucl Med Commun..

[105] van Waarde A, Jager PL, Ishiwata K, Dierckx RA, Elsinga PH (2006). Comparison of sigma-ligands and metabolic PET tracers for differentiating tumor from inflammation. J Nucl Med..

[106] Spaeth N, Wyss MT, Pahnke J, Biollaz G, Lutz A, Goepfert K (2006). Uptake of 18F-fluorocholine, 18F-fluoro-ethyl-L: -tyrosine and 18F-fluoro-2-deoxyglucose in F98 gliomas in the rat. Eur J Nucl Med Mol Imaging..

[107] Bruehlmeier M, Roelcke U, Schubiger PA, Ametamey SM (2004). Assessment of hypoxia and perfusion in human brain tumors using PET with 18F-fluoromisonidazole and 15O-H2O. J Nucl Med..

[108] Paolillo M, Serra M, Schinelli S (2016). Integrins in glioblastoma: Still an attractive target?. Pharmacol Res..

[109] Iagaru A, Mosci C, Mittra E, Zaharchuk G, Fischbein N, Harsh G (2015). Glioblastoma multiforme recurrence: an exploratory study of (18)F FPPRGD2 PET/CT. Radiology..

[110] Heddleston JM, Li Z, McLendon RE, Hjelmeland AB, Rich JN (2009). The hypoxic microenvironment maintains glioblastoma stem cells and promotes reprogramming towards a cancer stem cell phenotype. Cell Cycle..

[111] Yamamoto Y, Maeda Y, Kawai N, Kudomi N, Aga F, Ono Y (2012). Hypoxia assessed by 18F-fluoromisonidazole positron emission tomography in newly diagnosed gliomas. Nucl Med Commun..

[112] Perrone M, Moon BS, Park HS, Laquintana V, Jung JH, Cutrignelli A (2016). A novel PET imaging probe for the detection and monitoring of translocator protein 18 kDa expression in pathological disorders. Sci Rep..

[113] Albert NL, Unterrainer M, Fleischmann DF, Lindner S, Vettermann F, Brunegraf A (2017). TSPO PET for glioma imaging using the novel ligand 18F-GE-180: first results in patients with glioblastoma. Eur J Nucl Med Mol Imaging..

[114] Sandström M, Velikyan I, Garske-Román U, Sörensen J, Eriksson B, Granberg D (2013). Comparative biodistribution and radiation dosimetry of 68Ga-DOTATOC and 68Ga-DOTATATE in patients with neuroendocrine tumors. J Nucl Med..

[115] Kiviniemi A, Gardberg M, Kivinen K, Posti JP, Vuorinen V, Sipilä J (2017). Somatostatin receptor 2A in gliomas: association with oligodendrogliomas and favourable outcome. Oncotarget..

[116] Hofman MS, Lau WFE, Hicks RJ (2015). Somatostatin receptor imaging with 68Ga DOTATATE PET/CT: clinical utility, normal patterns, pearls, and pitfalls in interpretation. Radiographics..

[117] Natarajan A, Mayer AT, Xu L, Reeves RE, Gano J, Gambhir SS (2015). Novel Radiotracer for ImmunoPET Imaging of PD-1 Checkpoint Expression on Tumor Infiltrating Lymphocytes. Bioconjug Chem..

[118] Tavaré R, McCracken MN, Zettlitz KA, Salazar FB, Olafsen T, Witte ON (2015). Immuno-PET of murine T cell reconstitution postadoptive stem cell transplantation using Anti-CD4 and Anti-CD8 Cys-Diabodies. J Nucl Med..

[119] Di Gialleonardo V, Signore A, Willemsen ATM, Sijbesma JWA, Dierckx RAJO, de Vries EFJ (2012). Pharmacokinetic modelling of N-(4-[18F]fluorobenzoyl)interleukin-2 binding to activated lymphocytes in an xenograft model of inflammation. Eur J Nucl Med Mol Imaging..

[120] Deri MA, Zeglis BM, Francesconi LC, Lewis JS (2013). PET imaging with ^89^Zr: from radiochemistry to the clinic. Nucl Med Biol..

[121] Nduom EK, Wei J, Yaghi NK, Huang N, Kong L-Y, Gabrusiewicz K (2016). PD-L1 expression and prognostic impact in glioblastoma. Neuro-oncology..

[122] Hettich M, Braun F, Bartholomä MD, Schirmbeck R, Niedermann G (2016). High-resolution PET imaging with therapeutic antibody-based PD-1/PD-L1 checkpoint tracers. Theranostics..

[123] Mayer AT, Natarajan A, Gordon SR, Maute RL, McCracken MN, Ring AM (2017). Practical immuno-PET radiotracer design considerations for human immune checkpoint imaging. J Nucl Med..

[124] Heskamp S, Hobo W, Molkenboer-Kuenen JDM, Olive D, Oyen WJG, Dolstra H (2015). Noninvasive imaging of tumor PD-L1 expression using radiolabeled Anti-PD-L1 antibodies. Cancer Res..

[125] Kikuchi M, Clump DA, Srivastava RM, Sun L, Zeng D, Diaz-Perez JA (2017). Preclinical immunoPET/CT imaging using Zr-89-labeled anti-PD-L1 monoclonal antibody for assessing radiation-induced PD-L1 upregulation in head and neck cancer and melanoma. Oncoimmunology..

[126] Bensch F, van der Veen EL, Lub-de Hooge MN, Jorritsma-Smit A, Boellaard R, Kok IC (2018). 89Zr-atezolizumab imaging as a non-invasive approach to assess clinical response to PD-L1 blockade in cancer. Nat Med..

[127] Broos K, Keyaerts M, Lecocq Q, Renmans D, Nguyen T, Escors D (2017). Non-invasive assessment of murine PD-L1 levels in syngeneic tumor models by nuclear imaging with nanobody tracers. Oncotarget..

[128] Donnelly DJ, Smith RA, Morin P, Lipovšek D, Gokemeijer J, Cohen D (2018). Synthesis and biologic evaluation of a novel 18F-labeled adnectin as a PET radioligand for imaging PD-L1 expression. J Nucl Med..

[129] Chakraborty AK, Weiss A (2014). Insights into the initiation of TCR signaling. Nat Immunol..

[130] Tavaré R, McCracken MN, Zettlitz KA, Knowles SM, Salazar FB, Olafsen T (2014). Engineered antibody fragments for immuno-PET imaging of endogenous CD8+ T cells in vivo. Proc Natl Acad Sci USA..

[131] Li A, Wu Y, Tang F, Li W, Feng X, Yao Z (2016). In vivo magnetic resonance imaging of CD8+ T lymphocytes recruiting to glioblastoma in mice. Cancer Biother Radiopharm..

[132] Antonios JP, Soto H, Everson RG, Moughon D, Orpilla JR, Shin NP (2017). Immunosuppressive tumor-infiltrating myeloid cells mediate adaptive immune resistance via a PD-1/PD-L1 mechanism in glioblastoma. Neuro-oncology..

[133] Morisse MC, Jouannet S, Dominguez-Villar M, Sanson M, Idbaih A (2018). Interactions between tumor-associated macrophages and tumor cells in glioblastoma: unraveling promising targeted therapies. Expert Rev Neurother..

[134] Bingle L, Brown NJ, Lewis CE (2002). The role of tumour-associated macrophages in tumour progression: implications for new anticancer therapies. J Pathol..

[135] Zhang C, Yu X, Gao L, Zhao Y, Lai J, Lu D (2017). Noninvasive imaging of CD206-positive M2 macrophages as an early biomarker for post-chemotherapy tumor relapse and lymph node metastasis. Theranostics..

[136] Yang R, Sarkar S, Yong VW, Dunn JF (2018). In vivo MR imaging of tumor-associated macrophages: the next frontier in cancer imaging. Magn Reson Insights.

[137] Melancon MP, Lu W, Huang Q, Thapa P, Zhou D, Ng C (2010). Targeted imaging of tumor-associated M2 macrophages using a macromolecular contrast agent PG-Gd-NIR813. Biomaterials..

[138] Liao W, Lin J-X, Leonard WJ (2013). Interleukin-2 at the crossroads of effector responses, tolerance, and immunotherapy. Immunity..

[139] Wu C, Li F, Niu G, Chen X (2013). PET imaging of inflammation biomarkers. Theranostics..

